# Delivery of Neuropsychological Interventions for Adult and Older Adult Clinical Populations: An Australian Expert Working Group Clinical Guidance Paper

**DOI:** 10.1007/s11065-023-09624-0

**Published:** 2023-11-30

**Authors:** Dana Wong, Kerryn Pike, Rene Stolwyk, Kelly Allott, Jennie Ponsford, Adam McKay, Wendy Longley, Pascalle Bosboom, Antoinette Hodge, Glynda Kinsella, Loren Mowszowski

**Affiliations:** 1 School of Psychology and Public Health, La Trobe University, Melbourne, Australia; 2 School of Psychology and Public Health & John Richards Centre for Rural Ageing Research, La Trobe University, Melbourne, Australia; 3 School of Applied Psychology, Griffith University, Gold Coast, Australia; 4 School of Psychological Sciences, Turner Institute for Brain and Mental Health, Monash University, Melbourne, Australia; 5 Monash-Epworth Rehabilitation Research Centre, Melbourne, Australia; 6 Orygen, Parkville, Australia; 7 Centre for Youth Mental Health, The University of Melbourne, Parkville, Australia; 8 MERRC, Rehabilitation and Mental Health Division, Epworth HealthCare, Richmond, Australia; 9 Rehabilitation Studies Unit, Sydney Medical School, University of Sydney, Sydney, Australia; 10 The Uniting War Memorial Hospital, Waverley, Sydney, Australia; 11 MindLink Psychology, West Perth, Australia; 12 School of Psychological Science, University of Western Australia, Crawley, Australia; 13 The Children’s Hospital at Westmead, Westmead, Australia; 14 Faculty of Science, School of Psychology & Brain and Mind Centre, The University of Sydney, Sydney, Australia

**Keywords:** Neuropsychological intervention, Cognitive rehabilitation, Clinician competencies, Clinical implementation

## Abstract

Delivery of neuropsychological interventions addressing the cognitive, psychological, and behavioural consequences of brain conditions is increasingly recognised as an important, if not essential, skill set for clinical neuropsychologists. It has the potential to add substantial value and impact to our role across clinical settings. However, there are numerous approaches to neuropsychological intervention, requiring different sets of skills, and with varying levels of supporting evidence across different diagnostic groups. This clinical guidance paper provides an overview of considerations and recommendations to help guide selection, delivery, and implementation of neuropsychological interventions for adults and older adults. We aimed to provide a useful source of information and guidance for clinicians, health service managers, policy-makers, educators, and researchers regarding the value and impact of such interventions. Considerations and recommendations were developed by an expert working group of neuropsychologists in Australia, based on relevant evidence and consensus opinion in consultation with members of a national clinical neuropsychology body. While the considerations and recommendations sit within the Australian context, many have international relevance. We include (i) principles important for neuropsychological intervention delivery (e.g. being based on biopsychosocial case formulation and person-centred goals); (ii) a description of clinical competencies important for effective intervention delivery; (iii) a summary of relevant evidence in three key cohorts: acquired brain injury, psychiatric disorders, and older adults, focusing on interventions with sound evidence for improving activity and participation outcomes; (iv) an overview of considerations for sustainable implementation of neuropsychological interventions as ‘core business’; and finally, (v) a call to action.

## Introduction

### Background

In Australia, and many other countries around the world, the role of the clinical neuropsychologist in diagnostic assessment has long been emphasised, recognising the specialised skills neuropsychologists bring to identifying the presence, extent, and impact of cognitive and psychosocial sequelae associated with brain conditions across the lifespan. However, given its fundamental origins in human psychology and behaviour, its biopsychosocial approach to understanding individuals with brain conditions, and its emphasis on evidence-based practice, clinical neuropsychology also provides an invaluable framework for developing, providing, and evaluating interventions that target cognitive, emotional, behavioural, and psychosocial difficulties to improve overall functioning. New technologies that assist with diagnosis of brain conditions have led to a changing role of clinical neuropsychologists, with an increasing focus on the delivery of effective interventions.

Despite a long history of research and practice in neuropsychological interventions (Brouwer, 2002; Ponsford et al., 1995; Zangwill, 1947) and a quickly growing evidence base (Wilson, 2017), this aspect of clinical neuropsychological practice remains under-recognised among medical specialists, allied health practitioners, health policy-makers, and the lay public in many Australian settings (exemplified by the Stroke Foundation’s, 2020 Rehabilitation Services audit: https://informme.org.au/stroke-data/Rehabilitation-audits). This limits our ability to optimally contribute to patient care and management. Many neuropsychologists in Australia lack confidence in delivering neuropsychological interventions, at least partly due to limited opportunities for supervised practice during postgraduate training (Wong et al., 2014). These factors have led to gaps in the understanding and delivery of neuropsychological interventions in Australian clinical settings, which limits access to evidence-based care for individuals with brain conditions (e.g. Andrew et al., 2014; Naismith et al., 2022). We hope that this clinical guidance paper will represent one step towards addressing this lack of access. In response to changes to the opportunities available for funding neuropsychological interventions in Australia (e.g. the National Disability Insurance Scheme) and in order to open up further funding avenues (e.g. Medicare), it is imperative that we have high quality evidence for these interventions, and that our training programs are equipping clinical neuropsychologists with the competencies necessary to deliver evidence-based interventions effectively.

### Aims

This clinical guidance paper aims to provide an overview of key considerations in the effective delivery of neuropsychological interventions; clinical competencies important for intervention delivery; and clinical implementation of evidence-based interventions, alongside a summary of available evidence for their efficacy in the following populations:

- Acquired brain injury/illness (including stroke, traumatic brain injury, and multiple sclerosis);
- Psychiatric conditions (including early psychosis, schizophrenia, bipolar disorder, depression and eating disorders);
- Older adults (ranging from healthy ageing through mild cognitive impairment to dementia).

We acknowledge that this is not an exhaustive list of the potential groups with which neuropsychological interventions can be conducted; however, both research and practice are the most prominent in these cohorts. The evidence-based practice principles outlined both generally and for these specific groups are likely to apply to interventions delivered with people with other primary diagnoses (e.g. adult ADHD), although clinicians are encouraged to explore the evidence base for these specific cohorts to determine which approaches or techniques may be most appropriate and effective. Pragmatically, and in recognition of the unique clinical, psychosocial, and environmental parameters of relevance to interventions in children and adolescents, only neuropsychological interventions conducted with adult and older adult populations will be included in this clinical guidance paper.

We do not aim to provide a systematic or meta-analytical review of the evidence, as in many cases, this has been done previously. Where available, we will draw on and cite such existing reviews (see ‘Resources’ section for a list of key systematic reviews). We have also not performed a systematic appraisal of the quality of evidence for each intervention type, as would be expected for formal clinical guidelines (which is one of the reasons we have termed this a ‘clinical guidance paper’). This is because there is a large number of cognitive, psychological and behavioural interventions reviewed for each cohort, and to provide a systematic appraisal of evidence quality would be a very substantial undertaking that was beyond the scope of this paper. Rather, we have provided a synthesis of existing findings, particularly regarding the nature of available evidence for interventions with meaningful impacts on everyday life, as well as guidance and recommendations for a ‘best practice’ approach to incorporating these interventions clinically.

In doing so, we also aim to:

(i) Outline the role of clinical neuropsychologists in delivering neuropsychological interventions in Australia, while acknowledging interstate variability in training and roles within the public and private healthcare systems;
(ii) Highlight issues to consider in the planning and implementation of neuropsychological interventions for various patient groups;
(iii) Address key issues impacting the effectiveness of neuropsychological interventions, relating to the (a) client, (b) clinician, and (c) intervention technique;
(iv) Recognise the role and value of neuropsychological interventions in collaboration with other allied health and medical disciplines (e.g. occupational therapy, speech pathology).

We anticipate this paper may provide a starting point for future development of formal clinical guidelines regarding the use of neuropsychological interventions. At a broader level, we hope this paper may also provide an impetus for further collaborative and multidisciplinary research into developing, evaluating, and implementing neuropsychological interventions in Australia and internationally.

### Intended Audience

This clinical guidance paper is aimed primarily at clinical neuropsychologists with involvement, or intended involvement, in delivering neuropsychological interventions across a range of populations. It may also be useful for clinicians and researchers in other disciplines working within a multidisciplinary setting, where neuropsychological interventions may represent a component of a team-based therapeutic approach. In this case, the evidence and issues discussed herein may provide some context and parameters around the utility or expected outcomes from the neuropsychological intervention and how it may best be integrated alongside other medical or allied health interventions. Communication to our multidisciplinary colleagues about the potential benefits that neuropsychologists can offer was a critical need identified by Kubu et al. (2016); indeed, access to guidance papers detailing the value of neuropsychology was one of their specific recommendations.

We acknowledge that this paper has been written in the Australian context and that some issues (particularly relating to training, work roles, or funding) may differ in other countries. We have acknowledged these differences wherever relevant throughout the paper. We welcome commentary and perspectives from international colleagues about how the contents of this paper may apply in other countries.

## Development and Methodology

The concept for this paper was initially raised by Professor Sharon Naismith, following a symposium she led on this topic at the annual conference of the Australian Psychological Society’s College of Clinical Neuropsychologists (CCN), held in Melbourne, Victoria, September 2016.

The proposal was supported and a working party convened, comprising a network of clinical neuropsychologists actively working or researching the development, evaluation, or implementation of neuropsychological interventions. With support from Professor Naismith, A/Prof Dana Wong and Dr Loren Mowszowski were voluntarily appointed as co-chairs. Potential working party members were identified through professional networks and invited to participate on a voluntary basis. An effort was made to engage members throughout Australia, while limiting the number of members to a maximum of 15, to maintain project feasibility.

Once convened, the working party communicated via emails and teleconferences to develop a proposed outline regarding the aims of the paper, the populations to include, and key additional issues to include in the discussion (see the ‘Aims’ section, above). Subsections were allocated on a voluntary basis and according to areas of specialised skill or expertise.

The proposed paper outline was presented at an Open Forum event to delegates of the CCN’s annual conference in Perth, Western Australia (November 2017). Approximately 40 people attended the Open Forum, mostly neuropsychologists. During the forum, the working party requested feedback, comments, and suggestions from attendees regarding the proposed aims and outline of the clinical guidance paper. This feedback was documented, subsequently relayed to working party members not in attendance, and the aims and outline were amended accordingly. We note that few amendments were requested.

Thereafter, the working party proceeded to draft the paper according to their allocated subsections. All working party members reviewed each subsection and the entire paper to ensure consensus, consistency, and completeness. In April 2018, a paper draft was forwarded to the CCN Executive Committee, who provided feedback in September 2018. The working party subsequently amended the paper taking into consideration these comments and any updates in the field. Following this iterative process, a second draft was distributed to the CCN Executive Committee and overseeing expert Professor Sharon Naismith in 2020–2021. A presentation was also made to over 100 people outlining the contents of the paper as part of a CCN webinar (though non-CCN members also attended) in 2021, and attendees were invited to provide feedback, which was constructive and positive. Further revisions were made by the working party in 2021, with the revised version distributed to the CCN membership (966 members, including student members) for feedback towards the end of 2021. Respondents to this consultation supported the accuracy, clarity, and organisation of the contents, issues covered, evidence base, and usefulness of the paper. Feedback from respondents was considered when preparing the final version. Although CCN members were consulted, the expert working group are responsible for the contents of the paper.

## Section 1: Characterising Neuropsychological Interventions

### Defining Neuropsychological Interventions

There are multiple ways in which ‘neuropsychological interventions’ could be defined. For this clinical guidance paper, we define a neuropsychological intervention as *an intervention that targets the cognitive, emotional, psychosocial, and/or behavioural consequences of conditions affecting the brain.*

There are several points we wish to note about this definition. Firstly, we have chosen not to define ‘neuropsychological interventions’ according to the type or content of the intervention, as these are numerous. However, for the purpose of this paper, we will classify neuropsychological intervention types into the following main categories:

(i) Psychoeducation (including feedback after neuropsychological assessment)
(ii) Cognitive remediation/rehabilitation (encompassing restorative and compensatory approaches)
(iii) Psychological therapies (e.g. cognitive behaviour therapy)
(iv) Behaviour management (e.g. positive behaviour support plans)
(v) Environmental modifications and supports

These categories are very similar to those used by Wong et al. (2014) in their survey about the experiences of Australian neuropsychology graduates in delivering neuropsychological interventions. However, we acknowledge there are many variations in the labelling of these intervention types. This is particularly true for cognitive remediation/rehabilitation, which can encompass cognitive training, stimulation, and management using compensatory strategies, among other things. Throughout this paper, where evidence is specific to a particular intervention type, descriptive details will be included for clarity. It is also worth noting that categorising interventions by type or technique may falsely imply that these intervention types are standalone or separate; however, many neuropsychological interventions incorporate content from several of these categories and may be delivered in conjunction with other interventions (e.g. speech pathology, occupational therapy, medical or lifestyle interventions).

We also acknowledge there are other types of interventions that may be used to improve cognition or brain function, including pharmacological interventions and brain stimulation approaches (e.g. transcranial magnetic stimulation). We consider these types of interventions to be outside the scope of this clinical guidance paper, given (i) these interventions are not usually delivered by or accessible to clinical neuropsychologists, and (ii) most of these interventions have little evidence supporting their impact beyond the impairment level — i.e. at the level of activity and/or participation (see the next point). If either the accessibility or evidence for these other types of interventions changes, they could be included in updates to this clinical guidance paper.

**Fig. 1:**
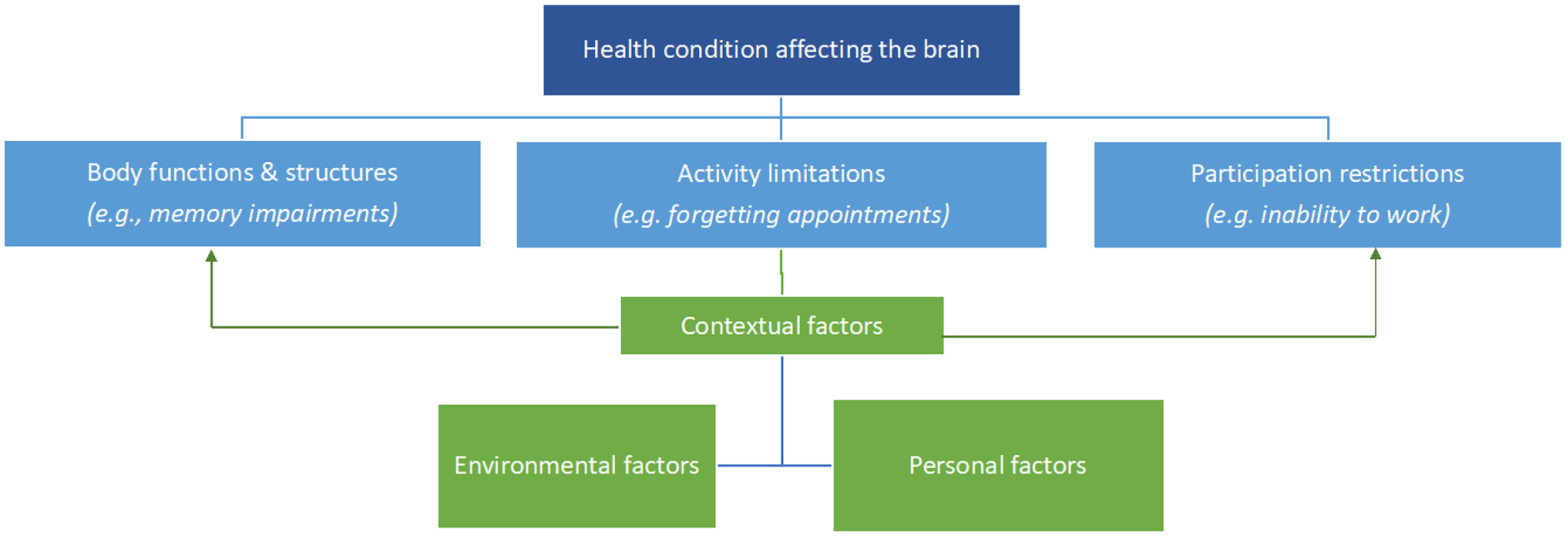
ICF model as applied to conditions affecting the brain, adapted from World Health Organization (2013)

Throughout the paper, we will use the International Classification of Function (ICF; World Health Organization, 2013) as a framework within which neuropsychological interventions can be understood and evaluated. In describing disability, this model distinguishes between ‘body functions and structure’ (e.g. impairment on memory tests), ‘activity limitation’ (e.g. forgetting appointments), and ‘participation’ (e.g. not being able to work due to memory difficulties), as shown in Fig. 1. Outcome evaluation often targets the impairment level, by measuring the impact of the intervention on test performance. However, we contend that the outcomes of neuropsychological interventions should also be evaluated in broader terms, that is, regarding their impact at the levels of activity limitation and participation restrictions and overall quality of life. Fundamentally, the aim of neuropsychological interventions is to improve everyday functioning to make a meaningful difference to the life of the person with the brain condition. The level of the ICF model at which outcomes have been evaluated will therefore be noted throughout.

### Delivering Effective Neuropsychological Interventions: Key Principles

While the evidence base for neuropsychological interventions has developed largely separately in different clinical populations, there are several key principles or considerations for the effective delivery of interventions that apply broadly. In this section, we introduce these general principles, which relate to the (a) client, (b) clinician, and (c) intervention.

#### Client-Related Factors

##### Using a Biopsychosocial Case Formulation Framework

**Fig. 2:**
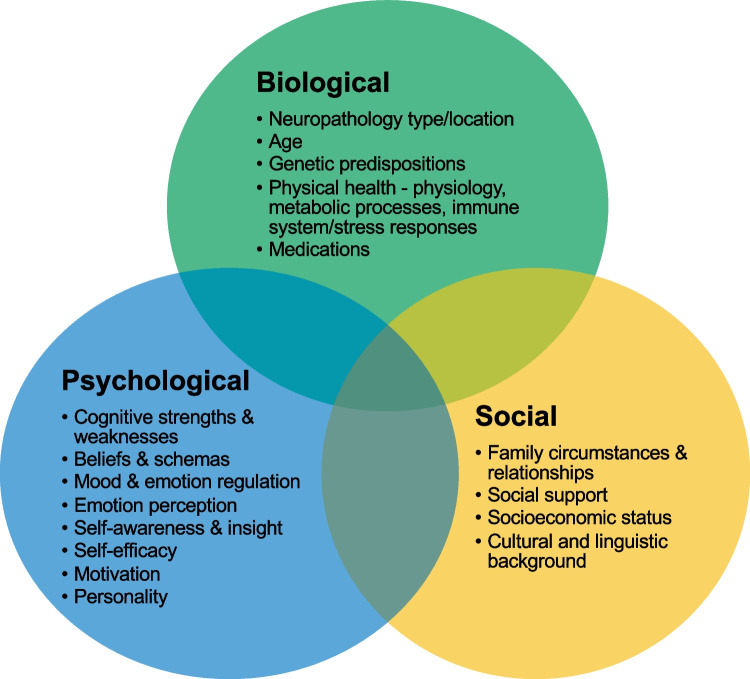
The biopsychosocial model of case formulation

Neuropsychological interventions should be founded on a comprehensive biopsychosocial case formulation considering biological, psychological, and social factors (Wilson, 2002) as depicted in Fig. 2.

A comprehensive assessment of all these factors is therefore strongly recommended when planning a neuropsychological intervention, so the intervention can be tailored accordingly. For example, consider the following individuals, referred for intervention targeting their everyday memory difficulties:

- John, a 39-year-old roof tiler, who had a severe traumatic brain injury (TBI) 6 months ago after falling from a ladder at work; has pre-existing sleep apnoea; lacks insight into his memory impairment; is apathetic with low levels of daily physical and mental activity; has low motivation to change; takes benzodiazepines more often than recommended; and whose wife works full-time while supporting their three young children and is very distressed;
- Sarah, a 23-year-old medical student, recently diagnosed with multiple sclerosis; has everyday memory difficulties that appear to be closely related to her attentional abilities and fatigue; has a history of anxiety and perfectionism; is highly motivated to finish her medical degree; and lives at home with her parents who are of Indian background and are resistant to the idea of Sarah seeing a psychologist;
- Brian, a 72-year-old farmer in a remote area, with possible early Alzheimer’s dementia; history of diabetes (with inconsistent medication adherence), cardiovascular disease, and depression; is the sole carer for his wife who has late-stage cancer; has been forgetting to feed the farm animals; is currently drinking at least four beers every night; distrusts doctors; has been told he should have a driving assessment and may lose his licence.

For each of these people, the content, timing, location, mode of delivery, sequence of intervention elements, and involvement of family in the memory intervention need tailoring according to their individual biopsychosocial formulation; as a one-size-fits-all approach is highly unlikely to be effective. For example, for John, behavioural and psychological interventions to address his apathy and motivational issues may need to be addressed prior to engaging him in memory rehabilitation strategies, while prioritising concurrent support for his wife (e.g. with a social worker). For Sarah, a focus on her study skills may enable engagement with a psychologist in the context of her family’s beliefs, and integrating fatigue and anxiety management strategies with memory interventions would be important. For Brian, establishing practical supports (e.g. through aged care services) to ensure the safety of his wife, farm animals, and other drivers would be of primary importance, along with memory and organisational strategies to ensure medication adherence.

##### Considering the Impact of Time Post-onset and Trajectory of Change

The nature of the neuropsychological intervention should also be tailored according to time since onset of the brain condition, and likely trajectory of the illness or injury. In the case of acquired brain injury (ABI), interventions in the first 6–12 months might focus on enhancing recovery and providing psychoeducation, with a later focus on managing and compensating for the residual effects of the ABI. In contrast, for mild cognitive impairment and dementia, in the early stages, interventions may focus on strategies used by the client themselves, whereas in later stages the interventions are likely to focus on family and environmental supports. For psychiatric disorders, interventions need to account for whether the mental illness is in an acute phase or remission and may also need to target issues such as risk of recurrence alongside improvement.

#### Clinician-Related Factors

##### Ensuring Clinicians Have the Appropriate Competencies

The clinician delivering the neuropsychological intervention should ensure they have the necessary competencies and resources to deliver the intervention effectively. This often involves additional training and/or supervised practice beyond that received in the context of university training programs. This topic is reviewed more comprehensively in ‘Section 2: Acquiring Competencies in Neuropsychological Interventions’.

##### Interdisciplinary Collaboration

Where possible, interdisciplinary approaches to management of individuals and families with brain conditions are recommended, throughout the continuum of care in hospital and community settings. This allows discipline-specific expertise to be integrated to work towards the client’s goals. Neuropsychological interventions are therefore often ideally delivered in an interdisciplinary or multidisciplinary context, together with other medical and allied health interventions and with the input of team members from other disciplines (Pagan et al., 2015) to determine priorities for targeted and timely intervention. For neuropsychologists working in private practice, there may be fewer opportunities to work within a multidisciplinary team. However, regular consultation and communication of treatment targets, plans, and progress to other health professionals involved in the person’s care (including the general practitioner or primary care physician) can be helpful in ensuring collaborative work towards common goals. Training that includes a multidisciplinary focus will provide team members with a necessary understanding of shared or complementary areas of practice in addition to more discipline-related expertise, assuring effective teamwork (Pagan et al., 2015).

#### Intervention-Related Factors

##### Intervention Targets Should Be Person-Centred and Goal-Directed

**Fig. 3:**
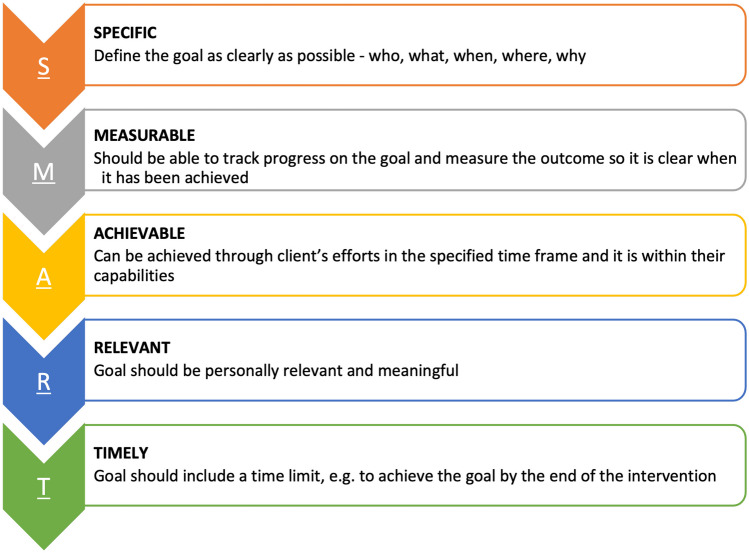
Goal setting using the SMART framework

Neuropsychological interventions should adopt a collaborative goal-setting approach, where person-centred goal(s) are set and refined as a key part of the intervention and progress towards the goal(s) is actively monitored. Goals should be set by the client; however, clinicians often need to assist clients in ‘unpacking’ their goals to ensure that the goals are SMART (outlined in Fig. 3).

Inevitably, family members, caregivers, and other stakeholders (e.g. employers) may also wish to contribute to goal development. While it can be difficult to navigate these potentially differing perspectives, the clinician’s role is to prioritise person-centred care. This includes balancing the needs of the client and ensuring they have adequate opportunity and means to communicate their wishes and opinions, while respectfully and constructively considering input from relevant others (in accordance with the biopsychosocial framework, as above). It can be helpful to define the roles of the treatment team and explain the person-centred approach to all involved parties at the outset of the intervention.

##### Interventions Should Be Evidence-Based

**Fig. 4:**
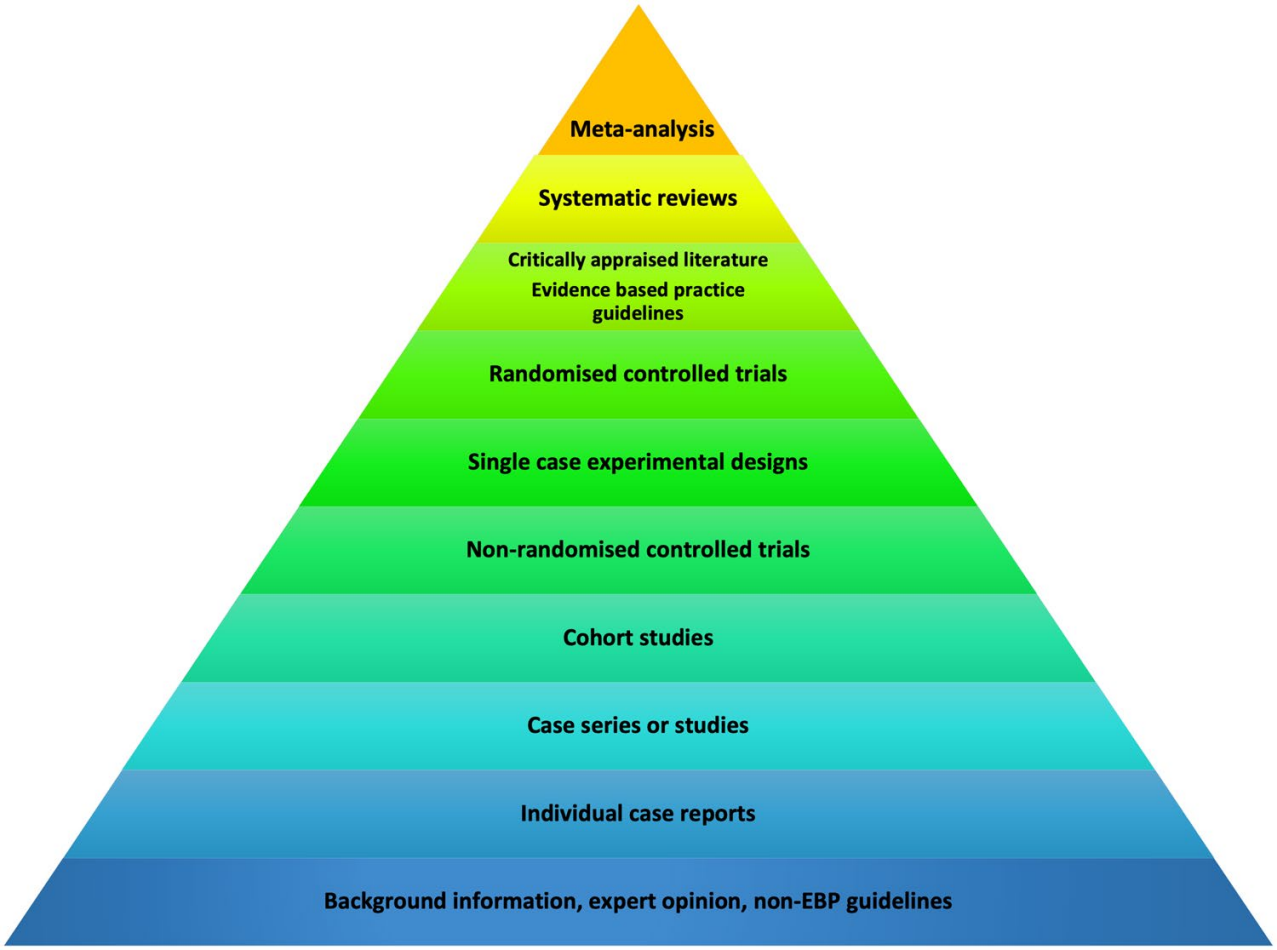
Hierarchy of evidence-based practice

Techniques and approaches with the strongest level of available evidence should be selected. Figure 4 shows the hierarchy of evidence that should be used to guide the choice of neuropsychological intervention.

In evaluating the evidence, clinicians should consider not just the strength and quality of the evidence for the overall *efficacy* of a particular intervention, but also:

- Known *predictors* or *moderators* of intervention outcome, and how these apply to the client in question;
- Whether or not the intervention outcome was *clinically significant* or meaningful, not just statistically significant; and
- Whether the outcomes were measured at the levels of *activity* and *participation* as well as impairment, given the overall aim of optimising quality of life.

Tools such as NeuroBITE (https://neurorehab-evidence.com/web/cms/content/home) and CogTale (https://cogtale.org/) can be useful resources to help clinicians evaluate the available evidence for particular interventions. It is important that clinicians can justify their choice of neuropsychological intervention based on the available evidence, the patient’s goals and preferences, their biopsychosocial context, and the clinical setting. As stated by van Heugten (2017, p. 22), ‘planning treatment explicitly and evaluating the outcome should therefore be a self-evident process, either by monitoring the individual patient or applying the best available evidence in a careful and judicious manner’.

##### Optimising Frequency/Duration/Intensity

In planning the neuropsychological intervention, consideration should be given to the frequency and duration of intervention sessions, and the total duration of the intervention period. Decisions about these factors should be based on available evidence for the efficacy of the intervention techniques under consideration, as well as in relation to the client’s circumstances, client goals, and feasibility (e.g. availability of clinicians, funding, geographical location of client, resources required to implement the intervention).

##### Choosing the Appropriate Context/Location and Mode of Delivery

Neuropsychological interventions targeting a particular presenting problem (e.g. attentional difficulties, social anxiety) can be delivered in a range of contexts and modalities. These include home-based or centre-based locations, individual or group formats, computerised or clinician-facilitated programs, and telehealth or face-to-face delivery. Again, decisions about the context and mode of delivery of a neuropsychological intervention should be based on the available evidence for that intervention type, as well as the client’s circumstances, goals, and feasibility (e.g. availability of services, technological proficiency of client, geographical location of client).

##### Enhancing Generalisability and Maintenance

Key elements of the effectiveness of a neuropsychological intervention include its ability to generalise to daily life roles and activities that have not been directly targeted in the intervention; and for its effects to be sustained beyond the period of intervention. Ideally, neuropsychological interventions that have demonstrated evidence of both generalisability and maintenance should be selected, and in delivering the intervention, clinicians should also actively discuss with clients how best to apply or incorporate the intervention techniques into their daily lives, both during and beyond the intervention period. Clinicians and researchers should also include some evaluation of generalisability and maintenance as part of their outcome measurement.

##### Using Meaningful Outcome Measures

Both clinicians and researchers should select appropriate measures to evaluate the outcomes of neuropsychological interventions, which are meaningful and relevant to client goals, as well as reliable and valid. Even if the intervention targets an impairment (i.e. at the bodily functions and structures level of the WHO ICF framework), the outcomes of the intervention should also be measured at the levels of activity and participation. This means including measures that capture everyday activities and participation in life roles such as work and leisure. Measuring goal attainment, for example using Goal Attainment Scaling (GAS), is a clinically useful way to capture progress on personally meaningful outcomes. Using GAS in addition to validated questionnaires measuring relevant activity and participation outcomes can together capture the outomes that are most important to the person. Furthermore, measures of health-related quality of life and wellbeing (which are not incorporated particularly well within the ICF framework) are also important and capture unique and important outcomes that can influence policy-makers deciding where to allocate resources. For measuring health-related quality of life, clinicians and researchers should be clear in selecting and reporting the perspective(s) used – i.e. self or carer. These are complementary perspectives and provide valuable information when used concurrently (Bosboom et al., 2012; Burks et al., 2021). Efforts should also be made by researchers to use consistent outcome measures when evaluating neuropsychological interventions, particularly those that target the same presenting issue or clinical population. Lists of recommended outcome measures have been compiled for adult TBI (Honan et al., 2017), brain impairment generally (Tate, 2010), and in the older adult field (Simon et al., 2020).

##### Consideration of Costs and Benefits

Clinicians and researchers should also consider the cost-effectiveness of neuropsychological interventions. These interventions are generally delivered in resource-constrained settings, whether funded through the government (e.g. primary health networks), insurance agencies, or the client. Therefore, the best interventions are not always those that have the best efficacy, if the margin of improved efficacy is slim but the cost increase is large compared with the next most effective intervention. In other words, a good neuropsychological intervention should deliver ‘bang for your buck’. Appraisal of costs and benefits can be particularly challenging where there may be several ways to deliver an intervention (e.g. group vs individual; face-to-face vs. telehealth), or where decisions must be made to use resources to deliver fewer interventions to more clients or more interventions to fewer clients. Cost-effectiveness is re-visited in ‘Section 4: Implementation of Neuropsychological Interventions into Health Services’.

## Section 2: Acquiring Competencies in Neuropsychological Interventions

### Which Competencies Are Required for Effective Delivery of Neuropsychological Interventions and How Should Clinical Neuropsychologists Be Trained?

A key challenge for the clinical practice of evidence-based neuropsychological interventions is training clinicians to be competent to deliver them effectively. This is one of the primary barriers to more widespread clinical implementation of interventions, with many neuropsychologists not feeling confident or competent enough to incorporate neuropsychological interventions into their clinical roles, at least in Australia (Wong et al., 2014). In their review of the implementation of evidence-based psychological treatments, McHugh and Barlow (2010) argue that the greatest challenge to dissemination is training clinicians who can competently administer these therapies. As they highlight, successful training of clinicians in evidence-based psychological therapies requires a balance of both *didactic* training (i.e. knowledge transfer through written materials and workshops) and *competence* training (i.e. acquiring the skills needed for delivering the treatment effectively, usually through supervised practice). The importance of training competent clinicians was also highlighted by Powell et al. (2012), in their review of strategies for implementing clinical innovations in mental health.

There have been several attempts to develop lists of foundational knowledge- and skill-based competencies for neuropsychological interventions (Rey-Casserly et al., 2012; Smith & CNS, 2018), and very recently, training pathways and competencies for ‘neurorehabilitation psychology’ (Stucky et al., 2023). In one of the most recent and comprehensive of these efforts, a group of American organisations calling themselves the Clinical Neuropsychology Synarchy (Smith & CNS, 2018) outlined five knowledge-based and seven applied intervention competencies. These CNS intervention competencies were recently further expanded by the Australian Neuropsychology Alliance of Training and Practice Leaders (ANATPL; Wong et al., 2023a). They indicated that clinical neuropsychologists should have *knowledge* of:

- Evidence-based intervention techniques and practices to address cognitive, emotional, and behavioural consequences of conditions affecting the brain, with consideration of both the quality of the evidence and whether there is evidence for meaningful impact on everyday activities, participation in life roles, and quality of life in the relevant clinical population.
- Theoretical and procedural bases of intervention methods appropriate to address disorders of attention, processing speed, learning and memory, executive skills, problem solving, language, perceptual and visuospatial skills, social cognition, psychological/emotional adjustment, and behaviours of concern.
- How complex neurobehavioural disorders (e.g. anosognosia, neuropsychiatric conditions) factors can affect the applicability of interventions.
- Sociocultural considerations when planning and using interventions, referring on to other providers with specialised competence if appropriate, and/or seeking cultural consultation as required.
- How to promote cognitive health with patients through activities such as physical exercise, cognitive stimulation, stress management, and healthy lifestyle (e.g. sleep, nutrition) practices.
- Empirically supported interventions provided by other psychologists and other mental and behavioural health professionals.

In terms of *applied* competencies, they indicated that clinical neuropsychologists should be able to:

- Identify targets of interventions and client goals and preferences.
- Employ neuropsychological assessment and provision of feedback for therapeutic benefit.
- Provide psychoeducation and information about neuropsychological disorders to aid the patient and family’s understanding of their presenting concerns and how to manage them.
- Identify potential barriers to intervention and adapt interventions to minimise such barriers.
- Develop a comprehensive biopsychosocial case formulation, including the cultural context, which usefully guides the intervention.
- Develop and implement treatment plans for neuropsychological problems based on the case formulation and client goals.
- Implement evidence-based cognitive interventions for neuropsychological disorders across the lifespan.
- Deliver evidence-based psychological therapies (e.g. for depression, anxiety) appropriately adapted for people with neuropsychological impairment.
- Provide behavioural interventions (e.g. positive behaviour support) for behaviours of concern in people with neuropsychological disorders.
- Consider suitability and provide adequate training and support for use of technologies within neuropsychological interventions (e.g. assistive technologies, telehealth).
- Independently evaluate the effectiveness of interventions employing appropriate outcome measures that are meaningful in everyday life, relevant to the patient’s goals, reliable and valid.
- Demonstrate an awareness of ethical and legal ramifications of neuropsychological intervention strategies.

While these foundational competencies apply generally to neuropsychological interventions, they do not describe how these competencies should be measured or benchmarked for specific intervention types. Many neuropsychological interventions require a unique and specialised set of skills that require specific training. It is therefore crucial to identify (1) which key competencies are required for effective delivery of each intervention, and (2) which methods for training clinicians are effective in enabling them to acquire those competencies. However, at present there is a paucity of evidence available to answer these questions. While there is a growing body of research on competencies and training in psychological therapies such as cognitive behavioural therapy (CBT) and motivational interviewing (MI), this work cannot necessarily translate to psychological therapies that have been adapted for individuals with neuropsychological impairments, given the additional skills required for those adaptations to be successful. For example, Roth and Pilling (2007) published a useful framework describing the competencies required for effective CBT for depression and anxiety; however, it does not contain any competencies for adapting CBT techniques for people with impairments in domains of cognition that are important for deriving benefit from CBT, such as memory, cognitive flexibility, self-monitoring, and planning.

In terms of training, combined workshop training and ongoing supervision improved both community therapists’ skills and client outcomes in CBT for depression (Simons et al., 2010), and a similar combination of training methods resulted in the greatest gains in proficiency in clinicians learning MI (Miller et al., 2004). Again, however, treatment recipients in both studies did not have cognitive impairments associated with brain injury or illness. When modified for people with ABI, CBT, when delivered with booster sessions, has been found to be effective in reducing anxiety and depression symptoms over a six-month period (Ponsford et al., 2016). This manualised intervention has been published by ASSBI resources (Wong et al., 2019b). Recently, Wong et al. (2020) evaluated the impact of workshop training and three sessions of clinical supervision of delivery of this intervention on the competencies of a sample of primarily neuropsychologists. They found that a mix of *didactic* and *competence* training resulted in self-rated improvements in adapted CBT competencies, which were maintained over a 16-month period. This type of work has potential to reduce barriers to workplace practice of adapted CBT relating to clinician competence.

Further research has been conducted to identify competencies and training methods for other neuropsychological interventions, including development of a checklist of competencies required for group-based rehabilitation interventions (Wong et al., 2019a) and neuropsychological assessment feedback (Wong et al., 2023b). However, this body of research is at its early stages and much more work is needed to establish the evidence to guide the training of clinicians who are competent to deliver neuropsychological interventions. Nevertheless, the evidence so far consistently indicates that training in neuropsychological intervention skills should actively incorporate both *didactic* training (focused on knowledge acquisition) and *competence* training (focused on skill development); and that the competencies required sit at the crossroads of clinical neuropsychology and rehabilitation/intervention delivery (Stucky et al., 2023).

### Current Training in Neuropsychological Interventions via Australian Clinical Neuropsychology Programs

At the time of writing and according to the Australian Psychology Accreditation Council (APAC) (www.psychologycouncil.org.au), training in clinical neuropsychology is offered by five accredited tertiary education providers across four states (New South Wales, Queensland, Victoria, and Western Australia). APAC provides accreditation standards for psychology programs (including Masters, PhD, Doctor of Psychology), which are approved by the Psychology Board of Australia (PsyBA), our national psychologist registration body. In the most recent iteration of the APAC guidelines (Australian Psychology Accreditation Council, 2019), which came into effect in January 2019, APAC is responsible for determining the accreditation of clinical neuropsychology training programs and have specified minimal content requirements for these programs. Intervention competencies for clinical neuropsychology programs (Section 4.1.3 of the APAC guidelines) comprise only:

- Selection, tailoring, and implementation of psychological interventions appropriate for clients and their needs, including rehabilitation, behaviour management, monitoring, and remediation;
- Consultation with and referral to other professionals regarding the neuropsychological implications of neurological and neuropsychiatric symptoms and disorders in a wider treatment context; and
- Psychological interventions appropriate to the behavioural and cognitive dysfunctions associated with neuropathology.

Previously, clinical neuropsychology competencies and accreditation were determined and overseen by the Australian Psychological Society’s (APS) *College Course Approval Guidelines for Postgraduate Specialist Courses* drafted in late 2010 and published on the Psychology Board of Australia website in January 2011 (Australian Psychological Society, College of Clinical Neuropsychologists, 2011). These guidelines offer more detailed guidance regarding proficiency in neuropsychological interventions, specifying formal knowledge in theories of recovery (e.g. neural recovery and reorganisation; functional adaptation) as well as evidence-based models and techniques of neuropsychological intervention for neuropsychological disability. Section 4.2.d of the 2010 College Course Approval Guidelines includes::

- Cognitive interventions for discrete cognitive impairments, such as visual neglect or memory disorder;
- Cognitive behavioural approaches (e.g. anger management);
- Psychotherapeutic approaches such as counselling;
- A ‘lifespan perspective’ allowing for moderation of principles and techniques across stages of life;
- Interdisciplinary teamwork and consultation; and
- Evaluation of the implementation and outcomes of any intervention in order to determine its efficacy.

This is nevertheless a clearly shorter list than the more detailed competencies for neuropsychological interventions recommended by ANATPL (Wong et al., 2023a). These accreditation guidelines should therefore be considered a minimum baseline for intervention competencies for graduates. In addition, while the content and format of accredited clinical neuropsychology training programs must comply with accreditation guidelines, there is currently considerable variability between states and education providers in terms of their methods for supporting neuropsychological trainees in developing the practical skills to plan, deliver, and evaluate neuropsychological interventions in clinical and/or research practice. Thus, currently, a postgraduate qualification in clinical neuropsychology does not necessarily signify an equivalent level of competency in neuropsychological interventions across graduates. Clinicians must always be aware of their own boundaries in competent practice (as clearly stated in our standards of ethical professional conduct) and seek additional training and supervision in areas of practice in which they have not had the opportunity to acquire the relevant skills.

Key areas of variability in training programs between states include:

(i) Proportion of course content devoted to intervention/rehabilitation, compared to diagnostic assessment and knowledge regarding various conditions affecting brain functioning;
(ii) Coursework content and formal supervised practice or skill development in counselling and psychological therapies (e.g. cognitive behavioural therapy, acceptance and commitment therapy, motivational interviewing, mindfulness, relaxation techniques).
(iii) Availability of clinical or research placements/internships where neuropsychological interventions are regularly implemented, in order to gain practical experience and develop skills in a supervised environment. Most placements tend to focus on developing and practising skills in diagnostic assessment, clinical formulation/interpretation, and communication of assessment outcomes, e.g. via reports and/or feedback.
(iv) Incorporation of opportunities to conduct or co-facilitate interventions within university training clinics. Students report positive feedback on these experiences when they are offered, describing increased skills and confidence in delivering interventions and an increased interest in conducting interventions in future (Pike et al., 2020).
(v) The inclusion of assessment tasks requiring students to demonstrate they can plan, implement, and evaluate neuropsychological interventions with real or simulated cases.

More consistent provision and evaluation of these training activities and models have the potential to enhance the breadth and depth of competencies in neuropsychological interventions among graduates of clinical neuropsychology training programs.

Ongoing training and upskilling beyond university training programs is necessary to ensure a skilled workforce, as reflected in the professional development requirements associated with psychology registration. Experienced clinicians may not have received comprehensive training or practice in delivering neuropsychological interventions and may have worked in settings where their role primarily involves assessment. Workshops, short courses, and clinical supervision may be beneficial for skill development and refinement in intervention skills and techniques throughout all career stages (Wong et al., 2021b, 2020).

While the structure of neuropsychology training differs around the world, these principles of ensuring neuropsychological intervention competencies, which have been clearly and comprehensively defined and are taught and assessed both during postgraduate training and beyond, apply internationally. Agreement on which competencies are essential for effective practice would facilitate international consistency in training and practice of neuropsychological interventions.

## Section 3: Evidence Base and Clinical Utility of Neuropsychological Interventions in Adults and Older Adults

In this section, we summarise key evidence and considerations for neuropsychological interventions in three main cohorts: (i) acquired brain conditions including traumatic brain injury (TBI), stroke, and multiple sclerosis (MS); (ii) psychiatric disorders; and (iii) older adults including healthy older adults, mild cognitive impairment, and dementia. In the Appendices, we summarise the evidence based on existing systematic reviews and guidelines in separate tables (labelled with ‘a’ and ‘b’ throughout):

- The interventions for each population for which there is sound evidence of impact at the level of activity/participation (for practical guidance on application of the WHO-ICF, see World Health Organization, 2013).
- Interventions for which sound evidence of impact on activity/participation is still required (i.e. evidence is at the level of impairment only OR there is only limited evidence of impact on activity/participation).

The evidence presented in the tables in the Appendices represents meta-analytic-level evidence where available or otherwise the highest level of evidence possible. We note the evidence will continue to build and expand, and we encourage clinicians to use the framework presented in the tables (i.e. classifying according to whether evidence of impact is at the level of activity/participation) to guide their clinical decision-making into the future.

### General Considerations

Across the three diagnostic cohorts, there is evidence that several types of neuropsychological interventions, particularly cognitive rehabilitation focused on personally meaningful goals and drawing on strategy-based training, can improve everyday function. There is limited support for functional or day-to-day benefits of computerised cognitive training as a standalone intervention when it is not combined with other (preferably personalised) support to apply strategies in everyday situations. Given that many computerised ‘brain training’ programs can require expensive subscriptions, clinicians should carefully seek high-level evidence of their efficacy and everyday impact before recommending them to clients. Brain training needs to be adaptive, intensive, and extensive for optimal restorative benefits. The SharpBrains checklist (Sharpbrains, n.d.) contains a good set of questions for consumers to ask themselves (or explore with their clinician) before embarking on a brain training program. Integrating health and lifestyle improvements (e.g. exercise, nutrition, and sleep) into neuropsychological interventions shows promise and clinical neuropsychologists are encouraged to incorporate evidence-based lifestyle recommendations in their feedback to clients. Group-based interventions also appear to have numerous benefits, including connection with others with similar lived experience. However, the ability to tailor interventions to individuals is a helpful advantage of those delivered one-on-one, and this is often necessary especially for people with complex needs.

There is some evidence that complex interventions that combine cognitive and psychological elements may be more effective in improving activity, participation and quality of life than treating impairments in isolation (Davies et al., 2023). The broader evidence for other combined or multicomponent interventions (e.g. combining behavioural with cognitive and/or psychological elements; combining restorative and compensatory cognitive training approaches; combining cognitive rehabilitation with physical or pharmacological interventions) is still developing and needs to be systematically evaluated across cohorts and intervention types. When considering whether to combine empirically supported intervention ‘modules’ into bespoke multicomponent interventions for individual clients, clinicians should consider not only the evidence for each component, but (i) also whether each component is justified by the case formulation, and (ii) the proposed underlying mechanism of action for each component, to ensure the combination of components has a solid rationale. The need for further research to help guide this kind of clinical decision-making is further detailed in the ‘Conclusions and Future Directions’ section.

### Neuropsychological Assessment Feedback

Often overlooked as a form of neuropsychological intervention, neuropsychological assessment feedback to the patient and caregivers can be conceptualised as its own brief, therapeutic, psychoeducational, single-session intervention. We cover it briefly here as an intervention that applies equally to all three diagnostic cohorts. Models or frameworks for delivering feedback have been proposed, but not yet thoroughly evaluated (Gorske & Smith, 2009; Postal & Armstrong, 2013). Evidence supporting the benefits of neuropsychological feedback is currently based primarily on consumer satisfaction surveys (Gruters et al., 2021). In one of the most recent surveys, neuropsychological assessment feedback was found to be followed by improvements in the patients’ quality of life, knowledge about their condition, and ability to cope (Rosado et al., 2018). A prospective follow-up study (Lanca et al., 2020) subsequently replicated and extended these findings, adding reduced psychiatric and cognitive symptoms, and increased self-efficacy and confidence in achieving goals to the list of benefits following feedback.

These findings contrast with concerns that a proportion of patients in these survey studies reported negative outcome following feedback (Longley et al., 2022), and that providing diagnosis-focused feedback (e.g. confirming likely dementia) can be harmful to some patients if not delivered sensitively. Reassuringly, a recent RCT with cross-over, in MS, showed no adverse psychological effects one week after feedback, despite most patients receiving ‘bad news’, and significant improvements in mood, self-efficacy, and perceived everyday cognitive functioning 1 month later (Longley et al., 2022). The authors outlined a list of feedback components they thought might have been psychologically protective for those participants receiving the bad news. We are also aware of upcoming trials of neuropsychological assessment and feedback as an intervention in dementia/mild cognitive impairment (MCI) and stroke. Collecting evidence for the therapeutic impact of feedback, as well as the most effective model of delivery, is an urgent and important priority for our field. It is often the first part of the neuropsychological intervention process and can be critical for further engagement. With supporting evidence, it could arguably be a primary neuropsychological intervention that all clinical neuropsychologists should consider delivering as a matter of routine practice.

### Common Neuropsychological Interventions

As many interventions have been trialled in all three of the populations discussed in this section, we have included a list of commonly used neuropsychological interventions that appear throughout the summaries and tables, briefly describing the nature and key features of each intervention. We note that this is not an exhaustive list of all existing neuropsychological interventions, and that the interventions listed here are not mutually exclusive. Where interventions have a high degree of overlap or have more than one label, we have indicated this in the table.

**Table:** 

**Adapted psychological therapies** *These aim to improve mental health and wellbeing in people with neuropsychological conditions and include adaptations to ensure therapy is suitable for people with cognitive impairment and/or neurological conditions*	
Acceptance and commitment therapy (ACT) — adapted for cognitive impairment	A psychological therapy that aims to change how an individual relates to distress and promotes engaging in behaviours consistent with the individual’s values. ACT components include the following: present moment awareness, acceptance, defusion, self-as-context, committed action, and values. Adaptations to ACT include reducing the more abstract components, and focusing on value-consistent behaviours
Cognitive behaviour therapy (CBT) — adapted for brain injury (CBT-ABI)	A psychological therapy that aims to identify and change unhelpful patterns of thinking and behaviour that contribute to the presenting issues. CBT components include the following: cognitive restructuring, behavioural activation, relaxation techniques, structured problem solving, graded exposure, and relapse prevention. Adaptations include use of repetition, simplified explanations and handouts, scaffolded cognitive restructuring, and greater emphasis on behavioural components
Compassion-focused therapy (CFT) — adapted for brain conditions	A psychological therapy promoting mental and emotional wellbeing by cultivating self-compassion. When applied to people with brain conditions, the focus of the self-compassion is on the impact of neuropsychological impairments
Mindfulness-based cognitive therapy	Combines cognitive behavioural techniques and mindfulness strategies to regulate thoughts and emotions and reduce distress related to the brain condition
**Behavioural strategies** *These aim to support individuals and families with managing behaviours of concern related to their brain condition*	
Anger management training	The use of cognitive behavioural techniques targeted at anger management concerns
Environmental modification	Aimed at reducing the impact of cognitive impairment or challenging behaviours by modifying the environment around a person to provide cognitive aids and/or remove potential triggers for challenging behaviour
Positive behaviour support (PBS)	A client-centred holistic approach that teaches new skills to replace behaviours that are challenging and optimise quality of life. By identifying and changing the antecedents and consequences of behaviours of concern, a positive behaviour support plan is developed with a focus on developing new skills that enable the person to do things that are meaningful to them
Social skills training	A form of behavioural therapy that covers a suite of interventions to improve social skills. Techniques include education and modelling appropriate behaviour, role play, corrective feedback, and positive reinforcement
**Cognitive strategies — compensatory** *These aim to minimise the impact of cognitive impairment on daily functioning through the use of aids or strategies to reduce cognitive load or perform affected tasks in a different way. Strategies include internal (mental) strategies, external aids (using tools in the environment), and systematic instructional approaches such as errorless learning. These are tailored to the cognitive strengths and weaknesses and personalised goals of the individual. Also can be termed ‘cognitive rehabilitation’*	
Attention process training (APT)	A range of tasks designed to exercise specific types of attention (sustained, selective, alternating, divided), administered in a hierarchical fashion (i.e. become more demanding)
Attention retraining	A range of exercises and compensatory strategies aimed at improving functional attentional abilities
Chaining	Involves breaking a task or procedure down into discrete steps. Each step is trained using prompts (verbal, visual, modelling), with gradual fading of prompts as the client becomes independent. Each step can be used as a prompt for the next step, so that the behaviours are ‘chained’ together. Both forwards and backwards chaining can be used. Training of each step often draws on an ‘errorless learning’ approach (see below)
Errorless learning	A method for systematically learning and remembering a novel task or skill. Tasks are broken down into small steps and the person receives immediate corrective feedback for each step to prevent them making a mistake (i.e. not trial and error learning). Helpful for those with intact procedural memory but impaired declarative memory
Goal management training	Aims to improve a person’s ability to complete purposeful everyday activities, usually in the context of executive dysfunction. Teaches a sequence of steps to raise awareness of attentional lapses, identify any problems, develop alternative solutions, monitor implementation, and check for errors
Group-based memory skills training	Group-based programs, usually of fixed length, that train use of internal and external memory strategies
Gesture training	Developing non-verbal communication skills to enhance communication for people with speech difficulties
Interpersonal process recall (IPR)	Aims to improve communication skills by using video playbacks of different social interactions between client and clinician. Clients are encouraged to provide self-feedback, in addition to the clinician’s feedback
Mental imagery	An internal compensatory strategy aimed at improving spatial neglect by asking the person to imagine in detail moving their body on the neglected side or attending to visual information on the neglected side
Metacognitive strategy training	Aims to improve performance of purposeful tasks through the use of various strategies to improve self-awareness, self-monitoring and self-correction of errors. Example strategies are self-talk, self-reflection and agendas to track progress
Modified story memory technique (mSMT)	Aims to improve episodic memory through the use of context and imagery
Music mnemonics	Aims to improve memory and recall by putting information to melodies
Retrieval practice	Aims to improve learning and memory by deliberately recalling information and actively ‘bringing it to mind’ repeatedly, as opposed to passively re-reading or listening to the information
Self-generation	Strategy to improve learning and memory in which people are given information with sections missing, and must generate answers themselves to fill the gaps (as opposed to being provided with all the information upfront)
Spaced retrieval (SR)	Aims to improve recall of information by eliciting active recall of newly learned information over progressively longer intervals of time
Strategy-based cognitive training (SCT)	Encourages the use of both internal techniques (e.g. visual imagery, categorisation, structured heuristics for problem-solving) and external techniques (e.g. calendars, environmental cues) to strengthen relevant cognitive functions and adapt to areas of weakness or decline by recruiting additional cognitive networks. SCT therefore involves active teaching, modelling and guidance in adaptive techniques by a facilitator. Many strategy-based approaches also incorporate psychoeducation regarding cognitive processes (e.g. encoding), which is often used to contextualise the strategies
Time pressure management	Consists of a set of compensatory cognitive strategies, deployed using a systematic process, to allow for mental slowness during real-life tasks by either preventing or managing time pressure
Video feedback	Direct corrective feedback may be used with people with impaired self-awareness. Approaches include involvement in contextualised occupationally-based activities, training to anticipate obstacles to optimise performance, verbal/audio-visual and experiential feedback, self-monitoring, and self-evaluation techniques
Visual imagery	An internal compensatory strategy aimed at improving memory for specific information by visualising it in rich detail
Visual scanning training	Encouraging people to actively pay attention (self-cue) to stimuli on a neglected side to improve visual scanning behaviour
**Cognitive strategies — restorative** *Aim to recover function in certain cognitive domains affected by brain injury by repetitive skill practice in the affected domain. Tasks are usually administered in a graded way, building on specific processes to become more complex*	
Cognitive remediation therapy (CRT) — includes computerised cognitive training (CCT)	Involves a broad range of learning-based interventions aimed at improving or restoring cognition broadly or in targeted domains. CRT typically involves repetitive drill-and-practice training exercises. CCT is the most common form of CRT, and involves specific computerised cognitive training programs aimed at improving particular aspects of cognition, commonly working memory. The software provides a structure in which to practice tasks, and is usually able to adapt task difficulty to suit ability. This can be paired with support from a clinician around strategies for improving task performance and how the training tasks and strategies are relevant and may be applied in daily life
Dual task training	Aimed at improving impaired executive functioning by practicing performing two competing tasks at once. Tasks can be any combination of cognitive and/or motor in nature
Eye patching	In the context of visual neglect, eye patching is used to encourage attention towards the neglected visual field by covering the intact visual field
Gist reasoning	Gist reasoning is the ability to abstract and generalise meaning from complex information. Cognitive training based on this top-down reasoning approach (as opposed to ‘bottom-up’ rote learning) aims to improve long term learning and fact recall
Mirror therapy	A body awareness intervention used in the rehabilitation of spatial neglect, focusing on proprioception and awareness of the body in space in relation to midline. A mirror is placed in the midsagittal plane to reflect the movements of one limb superimposed on the other limb
Perceptual interventions (e.g. sensory stimulation)	Aims to recover perceptual deficits. For example, sensory stimulation aims to stimulate visual sensation, such as shape recognition tasks
Strategic Memory Advanced Reasoning Training (SMART)	Aims to train functionally relevant complex reasoning abilities using a variety of control strategies (e.g. strategic attention learning to block out less relevant details)
**Other**	
Cognitive stimulation therapy	Promotes active cognitive stimulation and socialising, usually for people with mild to moderate dementia
Feedback	The provision of neuropsychological assessment feedback after testing is completed, usually at an additional session with the patient and/or family members. Information covered usually includes the patient’s cognitive strengths and weaknesses, psychoeducation on the likely causes for their presenting concerns (including diagnostic/biopsychosocial formulation), and management recommendations
Multimodal (or multicomponent) interventions	Interventions that draw on multiple components, often incorporating strategy-based CT, CCT, cognitive stimulation and psychoeducation; or combining cognitive, psychological and/or behavioural strategies. May also combine neuropsychological intervention with other forms of intervention, e.g. physical exercise, dietary supplementation, and medication
Psychoeducation	Refers to active communication and exchange of information to improve understanding about psychological, cognitive and/or behavioural issues associated with a brain or mental health condition. Information about the nature and causes of symptoms and how to manage or treat them is typically included. Can be delivered in individual or group formats
Social cognitive training	Includes a broad range of training exercises aimed at improving social cognition in everyday life. Social cognitive training typically involves engaging in therapist-facilitated exercises, and practice in applying the skills learnt to everyday social situations. These can include drill-and-practice exercises, strategy games, heuristic practice, mimicry, and role plays
Virtual reality training (VR training)	The use of virtual reality technology to aid motor and cognitive rehabilitation, usually by simulating scenarios in which a person performs a daily task such as cooking in a kitchen or going to the supermarket

### The Evidence for Neuropsychological Interventions in Acquired Brain Injury and Illness

The following section summarises evidence for neuropsychological interventions in acquired brain injury and illness (ABI), with a particular focus on traumatic brain injury (TBI), stroke, and multiple sclerosis (MS). While the ABI umbrella includes a range of other diagnostic groups including brain tumour, hypoxic brain injury, encephalitis, and epilepsy, the available evidence on interventions with these groups is more limited. However, research in this field often includes mixed samples, and many of the same principles apply across the various ABI types.

In some reviews, MS is placed in a group with other progressive neurocognitive conditions, such as Parkinson’s disease, where the focus of interventions might be to preserve cognitive functioning or delay the functional impact of neurobiological changes (Sumowski, 2015). However, in the context of our focus on activity and participation outcomes in this clinical guidance paper, intervention research on MS fits more readily with other forms of ABI, for whom stage of life and personal goals tend to be similar (e.g. sustaining paid employment by learning to retain new work instructions, or contributing to parenting via improved concentration).

Much of the existing evidence on neuropsychological rehabilitation interventions has been collected with ABI cohorts, particularly TBI. There is a wide range of effective cognitive, psychological, and behavioural interventions in these groups, plus a large selection of emerging interventions. The literature points to the importance of person-centred, goal-directed, tailored interventions that are meaningfully embedded in the person’s life roles and valued activities.

Clinical neuropsychologists are central members of multidisciplinary teams that support clients in their rehabilitation post-TBI. However, the healthcare system supporting survivors of stroke has evolved differently, particularly given the lack of insurance systems funding rehabilitation prior to the onset of the NDIS. Australian audit data (Stroke Foundation, 2020) found that over 50% of stroke services reported no access to a neuropsychologist. This is despite the clinical guidelines for stroke management (Stroke Foundation, 2017) supporting the need for intervention across multiple cognitive, emotional, and behavioural domains. There is an urgent need for support for a greater neuropsychology workforce in stroke services, to optimise outcomes for survivors of stroke.

Likewise, the neuropsychology workforce supporting people living with MS is not centralised in specific MS services and instead tends to be mostly private practitioners. People with MS without sufficient personal funds cannot access these sorts of services. This is despite a number of recent, clinically-focused overviews of the state of the science in this area (Brochet, 2021; Chen et al., 2021; DeLuca et al., 2020) which have described the evidence supporting a wide range of neuropsychological interventions in MS. For instance, DeLuca and colleagues (2020) concluded that ‘… cognitive rehabilitation has shown consistent beneficial effects in patients with MS and currently represents the best approach for treating MS-related cognitive impairment’. Consequently, there is also a urgent need for support for greater access to neuropsychology workforce in MS services (Longley, 2022).

#### Key Considerations

- The cognitive effects of TBI, stroke, and MS vary considerably from one individual to the next but may include impairments of attention and speed of information processing, memory, executive function, communication (including changes in social cognitive functions), and visuo-spatial function. Key principles that apply to addressing cognitive impairments in acquired conditions include the need to identify the individual’s pre-injury functioning and assessing the impact of problems in everyday contexts.
- For conditions that fluctuate or deteriorate rather than recover or improve, such as MS, there are additional psychological challenges for both the person and their caregivers, such as learning to live with an unpredictable combination of symptoms over time and an uncertain future. Some may also have to find ways of coping with distressing transitions (e.g. moving from a relapsing-remitting phase of MS to a secondary progressive phase, or moving from being independently ambulant with a walking aid to needing to use a wheelchair to get around). It should be noted that psychological therapies for depression, anxiety, and adjustment in MS have not been reviewed in this clinical guidance paper, since neuropsychologists generally do not deliver these interventions in Australia. However, there is a moderate and accumulating evidence base supporting therapies such as cognitive behaviour therapy in people with MS to treat depression (Khan & Amatya, 2017), stress and distress (Taylor et al., 2020), and fatigue (Harrison et al., 2021).
- Many TBI survivors are young and still in the process of establishing independence from parental support, studying or learning a vocation, and establishing important personal and social relationships. The inability to attain these important life goals can have devastating effects on their self-esteem and emotional state. Therefore, proactively addressing the cognitive, behavioural, social, and psychological barriers to attaining these goals is imperative. Their needs will change across the lifespan, so a long-term perspective is important.
- Due to the older demographic of many stroke survivors, it is important to advocate for access to rehabilitation to address goals such as independent living, work, caring, and leisure-related life roles. Compared to people who sustain an ABI in early adulthood, many older stroke survivors have established work and life skills and other resources that can be drawn upon to support their engagement in interventions. However, survivors are at an increased risk of developing dementia, particularly vascular dementia, Alzheimer’s disease (AD), or mixed vascular/AD (Savva & Stephan, 2010). Thus, ongoing monitoring of cognitive functioning is recommended to ensure the most appropriate neuropsychological interventions are provided over time.
- Multiple systematic reviews have highlighted a lack of high-quality studies investigating the efficacy of psychological therapies such as CBT in ABI (TBI/stroke) populations, which constrains the ability to provide firm recommendations (Fann et al., 2009; Gertler et al., 2015; Guillamondegui et al., 2011). Evidence for psychological therapies should be considered preliminary and clinicians are encouraged to monitor treatment progress to establish its efficacy in individuals (Gertler et al., 2015). In the absence of evidence for ABI specifically, psychological intervention is recommended if efficacy is demonstrated in the general population. However, psychological therapies such as CBT require modification for clients with ABI due to their cognitive impairments (Gallagher et al., 2019; Wong et al., 2019b).
- There is limited research evidence regarding the efficacy of antecedent or traditional consequence-based behaviour modification approaches to addressing behaviours of concern following ABI (including TBI, stroke, and MS). There is growing clinician support for contextualised approaches that modify the antecedents to behaviour problems based on consideration of precipitating factors that maintain negative behaviour, relating to the person, their injury-related cognitive impairments, and the environment, as well as factors that facilitate positive behaviour. Such approaches aim to assist individuals to self-manage behaviour, promote positive lifestyle changes, and increase community functioning and positive family environments. Personally meaningful activities and identified valued outcomes provide a basis for re-engagement with post-acute life (Ylvisaker et al., 2003). Positive, well-rehearsed routines are encouraged, to promote structure in and engagement with daily living. Feedback and consequences must be context sensitive and meaningful, and behavioural supports positive and proactive (Ylvisaker et al., 2007). Importantly, existing family members, friends, and carers are included in the adoption and integration of management techniques, and feedback is provided in the individual’s own environment, to encourage community-based and integrated supports (Ylvisaker et al., 2007). The integration of goal setting and community and clinical supports is important to achieving gains. An RCT of positive behaviour support (PBS) has shown that this approach can be effective in reducing challenging behaviours, with gains maintained over at least eight months post-intervention, in increasing the self-efficacy of close others in managing these behaviours (Ponsford et al., 2022), as well as attaining meaningful individual goals (Gould et al., 2021). A clinic has now been established and training resources for clinicians are under development using a co-design approach (Gould et al., 2019).
- For individuals with severe cognitive and behavioural difficulties following ABI, family members are an integral part of the support team. Recent evidence adopting a family-directed intervention approach shows promise in supporting the capability of family members to provide behaviour support (Fisher et al., 2021). The abovementioned recent RCT showed that a positive behaviour support intervention that actively involved close others including family (PBS-PLUS) resulted in a significant increase in their self-efficacy in managing challenging behaviours (Ponsford et al., 2022). However, much more research is needed.
- ABI is more common in indigenous Australians than in non-indigenous Australians; however, there is limited research evaluating culturally appropriate rehabilitation, and the research is particularly sparse when it comes to neuropsychological interventions (Lakhani et al., 2017). This is an urgent area for future research.
- The tables in this section have been separated into two Appendices, such that TBI, stroke, and other non-degenerative ABIs are included in Appendix 1 (Tables 1 and 2), and MS is included in Appendix 2 (Tables 3 and 4). This is because the MS literature is different in nature and scope, partly because MS is most commonly progressive, and much of the neuropsychological intervention research in MS has focused on specific techniques rather than the complex holistic interventions prominent in the TBI/stroke literature.

The evidence for the interventions presented in these tables is large and rapidly expanding. A number of systematic reviews and meta-analyses of RCTs or quasi-RCTs provide low-moderate evidence mostly at the impairment level (e.g. as measured by objective cognitive performance), but there is also growing evidence for positive activity, participation, and quality of life outcomes. There are numerous individual studies showing some benefits on these broader outcomes, but these findings may not yet have been replicated, so they have not been listed as providing sufficient evidence at the level of participation to warrant inclusion in Tables 1 and 2 (the literature on CBT for ABI is an example of this).

See Appendix 1 for a summary of the evidence in TBI, stroke and non-degenerative ABI (Tables 1 and 2). See Appendix 2 for a summary of the evidence in MS (Tables 3 and 4).

### The Evidence for Neuropsychological Interventions in Psychiatric Disorders

Neuropsychological interventions are critical for addressing functional recovery in adult psychiatric disorders, as neuropsychological (including social cognitive) impairments are common and often marked in psychiatric disorders (Abramovitch et al., 2021; McIntyre et al., 2019; Mesholam-Gately et al., 2009; Miskowiak et al., 2018; Semkovska et al., 2019). Such impairments are often evident early in the course of illness, even prior to the onset of full-threshold disorder, and are therefore considered to be core features of severe mental illnesses such as schizophrenia (Keefe & Fenton, 2007). Cognitive impairments tend to be persistent, including during periods of remission of other symptoms, and are more strongly associated with poorer daily living, social, and vocational functioning than mental health symptoms (Cowman et al., 2021; Fett et al., 2011; Lee et al., 2018). Accordingly, addressing cognitive impairment, is explicitly recommended in the Australian clinical practice guidelines for schizophrenia, other psychotic, and mood disorders (Galletly et al., 2016; Malhi et al., 2021). As neuropsychological interventions occur in the context of other mental health treatments, their prioritisation depends on the broader goals and preferences of the individual. Another consideration is the extent to which neuropsychological deficits prevent individuals from fully participating in and engaging with such mental health treatments. At a minimum, the neuropsychologist can provide psychoeducation to the client and their treating team regarding the role that cognitive impairment plays in hindering recovery goals and provide suggestions as to how this could be addressed in line with the individual’s priorities and incorporated with other treatments. Neuropsychologists take into account developmental, biological, psychological, environmental, social, behavioural, and treatment-related factors (both current and premorbid/developmental) when understanding neuropsychological profiles and prioritising areas for intervention, including, but not limited to, neurologically based disturbances, neurocognitive and social cognitive impairments, and social and daily living impairments. Cognitive strengths that are identified can be harnessed for promoting functional recovery and may help to address the commonly experienced motivational issues (Allott et al., 2020). In this cohort, consideration of the potential adverse effects of medication on cognitive function is also important (Albert et al., 2019), and may be dose dependent (Kasper & Resinger, 2003; Takeuchi, 2015).

#### Key Considerations

- The strongest evidence base for neuropsychological interventions is in schizophrenia and other psychotic disorders across the various clinical stages of illness (i.e. first episode through to non-remitting and chronic illness). These interventions are mostly grouped into computerised cognitive training (CCT) (Kambeitz-Ilankovic et al., 2019), cognitive remediation therapy (CRT) (Vita et al., 2021), or social cognition training (Nijman et al., 2020), but compensatory approaches are also well studied in psychotic disorders (Allott et al., 2020). There is emerging evidence for neuropsychological interventions in mood disorders (depression, bipolar disorder) (Bowie et al., 2013; Motter et al., 2016; Woolf et al., 2021) and eating disorders (particularly anorexia nervosa) (Tchanturia et al., 2014), given the growing recognition of cognitive impairments that impact on functional recovery in these conditions.
- Neuropsychological interventions within psychiatric populations typically target cognitive deficits and processes, with the goal of improving daily and community functioning. Interventions are commonly delivered in groups but may also be provided individually. Most neuropsychological interventions involve psychoeducation about cognition and/or social cognition and its relationship to the psychiatric condition as well as with daily functioning. Some neuropsychological rehabilitation programs are provided adjunctively with other psychosocial treatment programs (e.g. CBT or supported employment). Some studies have explored family/caregiver involvement to assist with implementation/adherence to the intervention outside of the immediate therapy environment (e.g. Kidd et al., 2018; Kumar et al., 2019), and this seems to be an area worthy of further research. Lifestyle interventions, including physical exercise and nutrition, are gaining traction in randomised controlled trials in psychiatric conditions, with growing evidence of benefits to cognitive function, particularly as adjunctive interventions (Firth et al., 2017).
- One issue that has impeded progress is that neuropsychological interventions have tended to be trialled in people with heterogeneous cognitive abilities and functioning in a ‘one-size-fits-all’ approach. There is growing recognition for the need to personalise neuropsychological interventions, with a large body of research examining predictors of treatment response, aiming to stratify or select participants based on particular characteristics (such as a particular degree of cognitive impairment and level of motivation). Recent reviews, however, have not been able to identify reliable predictors (i.e. demographic, biological, cognitive and functional, psychological, and illness-related characteristics) of response to cognitive remediation therapy in schizophrenia (Reser et al., 2019; Seccomandi et al., 2019), so the field still has some way to go to improve appropriate stratification of clients into cognitive rehabilitation trials.
- Nevertheless, recent efforts aim to identify the core components of effective cognitive remediation therapy within specific diagnostic groups to better guide future research and clinical practice that is personalised, effective, and meaningful to clients. Bowie et al. (2020) recently proposed four core components of cognitive remediation in psychosis as part of an expert working group which were found in a meta-analysis to positively moderate outcomes (Vita et al., 2021). These core components include the following: (1) involvement of trained cognitive remediation therapists, (2) multiple repetitions of cognitive exercises, (3) opportunities for participants to identify, use and monitor cognitive strategies, and (4) procedures to support transfer of trained skills to real-world functioning. This conceptualisation emphasises the importance of formulating cognitive problems and their relationship with functioning, capitalising on intrinsic motivation in the learning process, and modifying task difficulty to optimise success and collaborative, realistic goal setting and tracking (Bowie et al., 2020). Similar therapeutic considerations have been recently suggested for major depression (Douglas et al., 2020).
- For mood disorders, expert consensus statements focusing on cognition trial methodology have been proposed to guide the field in developing a strong evidence base for personalised neuropsychological interventions (Douglas et al., 2020; Miskowiak et al., 2017). Some of the key recommendations include the following: (1) ensuring adequate pre-screening to ascertain clients have cognitive impairment before enrolment; (2) enrolling clients when their mood is stable and relatively euthymic; (3) consideration of the effects of prescribed medication on the ability to benefit from the intervention and possibly optimise dosage (e.g. minimise use of benzodiazepines); (4) ensure outcome measures include co-primary or secondary measures of functioning (i.e. activity/participation), in addition to cognition; and (5) ensure participants receive an adequate dose (i.e. 10–20 weeks) of cognitive remediation therapy to maximise the intervention effects.
- While some cognitive remediation programs have been translated into languages other than English (e.g. Spanish, Arabic), we are not aware of published work that has directly addressed cultural sensitivity and/or adaptation in psychiatry (e.g. for Aboriginal and Torres Strait Islander peoples, or other culturally diverse groups), which is unfortunate and an area ripe for research and implementation.

See Appendix 3 for a summary of the evidence in psychiatric disorders (Table 5).

### The Evidence for Neuropsychological Interventions with Older Adults

There is an unfortunate tendency in the general community and among many health professionals, to underestimate or dismiss the utility of rehabilitation-focused interventions for cognitive decline in older adults. This is often due to misunderstandings including (a) that the underlying cause of all cognitive decline in older people is dementia, and (b) that therapeutic approaches in dementia are either unavailable, ineffective, or unrealistic given its progressive course (see discussion by Cations et al., 2018). However, clinical practice guidelines and current World Health Organisation recommendations for dementia care emphasise cognitive interventions among those rehabilitation approaches with the strongest evidence base (see Jeon et al., 2022; World Health Organisation, 2023), as there is sound evidence that neuropsychological interventions, particularly when individualised and goal-oriented, can improve everyday function in people with dementia. These interventions can maximise independence, delay the need for residential care, and result in important benefits for mood, quality of life, and meaningful participation in life roles such as grandparenting and community involvement.

Moreover, in those with MCI and even in older adults without objective cognitive impairment, there is sufficient evidence to support a recommendation from the World Health Organization (WHO) for the use of cognitive training to reduce the risk of cognitive decline and/or dementia (World Health Organization, 2019). In fact, these older adults with a higher risk of developing dementia are considered to represent an ideal population for early and selective prevention using cognitive interventions (Gates et al., 2014; Mowszowski et al., 2010; Pike & Kinsella, 2019), due to greater residual or compensatory cognitive resources, as well as greater insight. There is insufficient data to determine whether cognitive interventions have the capacity to *prevent* conversion to dementia (Butler et al., 2018), which at least partly relates to the methodological difficulties and expense in following participants for sufficiently long periods to gain these data (Huckans et al., 2013). Evidence in these groups indicates small improvements in cognition as well as some aspects of daily functioning following neuropsychological interventions. Given the small effects, tailoring an intervention to focus on individual goals is highly recommended. However, this does not mean that interventions should only be provided in a one-on-one format, as group interventions have several benefits, including peer support, opportunity for normalisation of experiences, and modelling.

A burgeoning area of clinical and research interest is the use of neuropsychological interventions for older adults without objective cognitive impairment, but who are concerned about a subjective decline in their cognition. There is mounting evidence that older adults with subjective cognitive decline have an increased risk for developing dementia and MCI (Mitchell et al., 2014; Pike et al., 2022). Furthermore, given that approximately 40% of dementia cases worldwide are attributable to potentially *modifiable* risk factors (Livingston et al., 2020), prevention of cognitive decline has become a particularly hot topic in recent years (Butler et al., 2018; Zokaei et al., 2017).

Indeed, a recent systematic *overview* of systematic reviews by Gavelin and colleagues (Gavelin et al., 2020) affirms the efficacy of cognition-oriented treatments across the continuum of cognitive presentations ageing. This rigorous review process synthesised quantitative findings for interventions spanning cognitive stimulation, training, and rehabilitation approaches across 46 previous systematic reviews and reported effect sizes of 0.3 to 0.4 for cognitive outcomes across healthy older adults, mild cognitive impairment, and dementia. While the systematic overview found that non-cognitive outcomes are unfortunately sparsely reported and recommended further research to more consistently evaluate clinical value across the field, we note that individual systematic reviews (e.g. Chandler et al., 2016) have reported effects on non-cognitive outcomes of clear clinical relevance (e.g. activities of daily living, mood, self-efficacy) and this is summarised in Appendices 4, 5, and 6.

It is also worth highlighting that among the dementia literature, few research studies have been conducted in non-AD or in young onset dementia (YOD). Considering that people with YOD are usually physically strong and healthy, often working at the time of diagnosis, may be supporting dependent children and/or ageing parents, and have significant financial obligations and commitments, the impact and burden of YOD can be great and often requires different types of support and services (Aplaon et al., 2017; Loi et al, 2023). There is some evidence that cognitive interventions improve functional outcomes and affective symptoms (Aplaon et al., 2017), but in general, the literature on YOD has limited methodological rigour (e.g. small sample sizes, few randomised controlled trials) (e.g. Fox et al., 2020) and thus is not included in our Appendices.

There is also evidence that health and lifestyle changes can positively impact cognition, with particularly strong evidence for the benefit of physical activity. Promising research is underway using technology to support memory in dementia, and we wait with anticipation for results of ongoing trials.

#### Key Considerations

- From a biopsychosocial perspective, significant issues frequently arise in this phase of life and may contribute to, or exacerbate, cognitive and/or day-to-day functioning. These include retirement, bereavement, adjustment and role change, mental health (e.g. depression, anxiety, loneliness), general health (e.g. chronic pain, diabetes), polypharmacy, sensory status (i.e. hearing, vision), and environmental context (e.g. independent living, residential care, or assisted living). It is critical to consider the impact of these concurrent factors on intervention planning and outcomes, as well as considering whether other, multidisciplinary approaches may assist in addressing these factors
- Psychological interventions play a key role in managing many of the highlighted factors, with important flow-on effects for cognition. Despite this, there is limited research on the cognitive effects of these interventions in older people, and psychological interventions are often delivered separately to neuropsychological interventions in this cohort. We propose that a more integrated approach to these intervention types may be optimal and may provide synergistic benefits and look forward to further research in this area (see the ‘Conclusions and Future Directions’ section).
- Family or other informal caregivers can play an integral role and should be involved and supported in the intervention process (Clare, 2017), but this must be balanced with upholding a person-centred approach. For example, avoiding (inadvertent or intentional) caregiver direction of intervention goals or selective adherence to intervention techniques. This balance can be facilitated by framing the caregiver as a ‘care partner’ in delivering the intervention and providing structured support and education regarding their role throughout (see, for example, the I-HARP trial; Jeon et al., 2019). Furthermore, caregivers of people with dementia often experience their own significant psychological, social, and financial impact. Here, neuropsychological interventions can address caregiver wellbeing including symptoms of depression and anxiety, as well as their ability to provide care, by providing psychoeducation to enhance understanding of changed cognition/behaviour and training in problem-solving, coping strategies, and behavioural management approaches (Bayly et al., 2021; Gilhooly et al., 2016).
- Older Aboriginal and Torres Strait Islander peoples have a higher prevalence of dementia, which has been linked with a variety of biomedical and sociocultural factors (e.g. male sex, childhood adversity and trauma, unskilled work, current smoking, head injury, and other chronic conditions) (see Li et al., 2014; Radford et al., 2015). Despite this, no research on neuropsychological interventions in this group has been published, and this is an important gap. Future research is critically needed and must incorporate collaborative work with Aboriginal and Torres Strait Islanders, such as a ‘collaborative yarning group’ (e.g. Lavrencic et al., 2021) study to help ensure feasible and culturally safe interventions.
- Many older people experience subtle cognitive changes as part of the *normal* ageing process. Some, however, experience more significant cognitive impairment relating to a variety of aetiological factors. Where neurodegenerative disease is a primary cause, cognitive impairments may be progressive, change in nature over time, and involve reduced awareness or insight. This does not mean that effective and meaningful therapeutic intervention is not warranted or cannot be achieved; rather, it again highlights the need for a considered and tailored approach to designing and implementing interventions for specific subgroups and individuals. Thus, we will consider separately (a) neurodegenerative diseases, with a focus on AD (as this is where most of the literature lies), (b) mild cognitive impairment (MCI), and (c) healthy ageing, including subjective cognitive decline.
- While most research in neurodegenerative disease is focused on AD, benefits of neuropsychological interventions in other less common types of dementia are emerging. Overall, an individualised approach is recommended to account for distinct symptom profiles (e.g. environmental, behavioural, and physical strategies for people with behavioural variant frontotemporal dementia and communication strategies for people with language variant frontotemporal dementia; see key resources for FTD at http://ecdc.org.au/ftd-toolkit.htm), and emotion recognition training in people with Huntington’s disease (see Kempnich et al., 2017).
- Notably, people with MCI may experience only subtle or minimal functional change due to cognition, while healthy older adults have no objective cognitive impairment or functional change due to cognitive impairment. As such, most studies in these subgroups focus solely or heavily on objective improvement in cognition (i.e. level of impairment) as a critical outcome, rather than measures reflecting activity or participation. In keeping with the overall approach of this clinical guidance paper, we have separately summarised the relevant literature with respect to effects at the levels of participation and activity (Tables 6 and 8) and impairment (Tables 7 and 9) for dementia and for MCI (Appendices 4 and 5, respectively). For healthy older adults, it should be noted that improvement in cognition, even in persons without cognitive impairment, is an important outcome that should not be dismissed (Table 10).

See Appendix 4 for a summary of the evidence in dementia (Tables 6 and 7).

See Appendix 5 for a summary of the evidence in MCI (Tables 8 and 9).

See Appendix 6 for a summary of the evidence in healthy older adults (Table 10).

## Section 4: Implementation of Neuropsychological Interventions into Health Services

The translation of research evidence on neuropsychological interventions (as reviewed in ‘Section 3: Evidence Base and Clinical Utility of Neuropsychological Interventions in Adults and Older Adults’) into widespread clinical practice is crucial to ensure improved outcomes can be achieved for individuals with brain conditions as part of their usual health care. The gulf between evidence and practice continues to be wide in many areas of health care (Bayley et al., 2012; Rogers, 2003), and it is incumbent upon clinicians, researchers, and health service managers to identify and apply methods for closing this gap to ensure consumers benefit from investment in research (Davis et al., 2003; Graham et al., 2006; Greenhalgh et al., 2004). This is a significant challenge for this field, given the traditional focus of the neuropsychologist’s role on assessment and diagnosis in Australia, which means that health services often do not have staffing and resources dedicated for clinical neuropsychologists to deliver interventions. For example, a recent national survey of memory clinics by the Australian Dementia Network (Naismith et al., 2022) indicated the limited provision of neuropsychological interventions even within these dedicated settings. Of the 55 memory clinics identified, only 20% provided memory strategy training; just two clinics (i.e. 3.6%) provided telehealth memory rehabilitation, and one (i.e. 1.8%) provided computerised cognitive training. Only 14.5% provided more than one session of a neuropsychological intervention, and even these were often limited to just two sessions. Group-based programs focused on cognition and wellbeing were provided by 12.7%. This is despite 74.4% of memory clinic respondents identifying neuropsychological intervention as a key ingredient of adequate post-diagnostic care.

Despite this appetite for additional intervention services, any staffing increases in resource-constrained health services require strong advocacy and evidence that the additional service provision will address a significant unmet need and potentially reduce future downstream costs. Collaboration with health economists can be extremely useful in providing an analysis of the cost-effectiveness of a new intervention, which is often crucial evidence for inclusion in a business case. Additionally, the involvement of and advocacy from consumers, particularly in highlighting the need for and benefit of the intervention, is important to health service managers whose job is to meet the needs of their consumers.

In the current climate of increasing pressure on healthcare resources, a key challenge is to demonstrate to funding agencies, healthcare services, and clients that neuropsychological interventions are not only effective, but also represent value for money (Prigatano & Morrone-Strupinsky, 2010; Stolwyk et al., 2021; Worthington et al., 2017). Health economic analyses include cost–benefit analysis (measuring the outcomes of an intervention in monetary terms such as productivity), cost-effectiveness analysis (measuring outcomes in terms of natural or health units such as days off work or health appointments), and cost-utility analysis (measuring outcomes in terms of quality-adjusted life years). Worthington et al. (2017) and Stolwyk et al. (2021) are helpful sources of further information about these concepts. These analyses can be used to demonstrate both the costs and benefits of various interventions which can inform clinical practice, service implementation, and the business case for rehabilitation programs or interventions. For example, van Heugten and colleagues (2011) reported cost savings from a societal perspective associated with participation in a community reintegration program following acquired brain injury. Using these data, the authors could argue that the cost of the program would be recouped after 8 years and therefore health insurance companies ought to reimburse these costs in view of the potential for long term savings with individuals having an average of 35 years to live before being eligible for pension support. In another example, Radford and colleagues (2013) demonstrated that individuals with traumatic brain injury were more likely to return to work following a vocational rehabilitation group, at a marginally increased cost compared to usual care. However, once the societal costs (e.g. loss of wages, expenses, employer costs) were considered, the vocational intervention was more cost-effective with it costing £12,418.20 (more than AUD 22,000) less to return a person to work with the intervention than usual care. These studies illustrate how clinical neuropsychologists and research teams can collaborate with health economists to justify the implementation of neuropsychological interventions within clinical rehabilitation practice.

Several theoretical frameworks are available to guide clinical translation and implementation efforts, including the Knowledge to Action Framework (Graham et al., 2006), the Consolidated Framework for Implementation Research (Damschroder et al., 2009), and the RE-AIM Framework (Glasgow et al., 1999). Common features of these frameworks include (i) identifying barriers and enablers, (ii) selecting intervention components and adapting them for the local context, and (iii) systematically monitoring the outcomes of implementation. Based on a review of strategies for implementing health innovations (Powell et al., 2012) and evidence from specific evaluations of neuropsychological intervention implementations (Kinsella et al., 2020; Wong et al., 2021b), the following key strategies for clinical implementation initiatives are recommended in Box 1.

## Box 1 Key strategies for clinical implementation of neuropsychological interventions

**Table:** 

**Plan and build engagement** (Allow plenty of time for this)	Discuss the goals of the organisation in implementing the new service — build ‘buy-in’ Develop the relationships necessary for successful implementation Engage service users/consumers as essential stakeholders Help stakeholders gather data Identify the organisation service delivery team and available staffing resources, and initiate leadership Restructure existing professional roles if necessary Identify a *Change Champion* in the organisation for leadership in delivery of the intervention
**Educate stakeholders**	Inform all relevant stakeholders about the innovation
**Tailor the intervention to the setting**	Determine the core components in the intervention that cannot be altered, and communicate these clearly to clinicians Be prepared to be flexible in responding to feedback from clinicians Adapt and adjust the non-core elements of the intervention
**Establish a training approach that maintains intervention fidelity**	Fit the training model to the capabilities and resources of the service Decide on strategies to maintain fidelity of the intervention (e.g. provide a detailed manual; provide opportunities for new clinicians to observe the intervention live or via video; provide supervision during initial stages of the roll-out) — this should encompass both *adherence* (to the intervention content) and *competence* (in its delivery)
**Secure finance**	Seek relevant funds/grants to finance the implementation Leverage existing funds to incentivise the use of clinical innovations and provide resources for training and ongoing support
**Evaluate the implementation to ensure quality management**	Agree on measurable intervention goals, and the available resources for evaluation Identify/develop outcome measures that are sustainable and appropriate to the organisation Evaluate outcomes for clients, clinicians, and the organisation Pilot, trial, and regularly review
**Examine sustainability**	Use the results of the outcome evaluation to identify costs and benefits Develop a plan for sustainability, including managing attrition of trained clinicians Encourage promotion of the innovation through hospital networks, foundations, events and media Proactively disseminate knowledge about the program to policy-makers to seek new funding arrangements

These strategies were effectively utilised in several recent Australian examples in which new neuropsychological interventions were introduced into public health services. In the MemoRI project (Wong et al., 2021b), a memory skills group program was implemented at two health services in which clinical neuropsychologists were trained and supervised in group facilitation. Subsequently, in collaboration with health economists, the cost-effectiveness of the new pathway of care was evaluated along with participant outcomes. The memory group was found to be clinically effective and more cost effective than standard care, and consumers and referrers provided strong feedback in support of the program. This information was used to support a business case for staffing increases/restructure at the two health services. That project was then replicated at several other health services in Victoria. In a separate initiative, Stolwyk and colleagues (in preparation) demonstrated the successful development and implementation of a pilot teleneuropsychology stroke rehabilitation (TNSR) service, delivered to a rural stroke rehabilitation unit from a metropolitan hub. Implementation of TNSR resulted in improved clinician competence and service quality in the provision of assessment and treatment for mood and cognitive dysfunction, with positive experience of the service reported from both patients and clinicians. Economic simulations demonstrated TNSR could be delivered more cost-effectively compared to an equivalent in-person service. This evidence was incorporated into a business case which supported ongoing funding for the service in addition to planned scale-up to multiple rural hospital sites.

Similar work is currently underway at St Vincent’s Hospital in Sydney and demonstrates the lengthy and complex process that often contributes to the evidence-to-practice lag. Commencing in 2017, an evidence-based cognitive training and psychoeducation program (see Diamond et al., 2015) was implemented within this metropolitan public health service for older outpatients with late-life depression. An initial pilot feasibility study was undertaken to iteratively adapt the intervention to suit the priorities and needs of patients and staff within the setting, including staff training and acquiring resources for the intervention. Having shown that the pilot implementation was feasible, well-tolerated, and highly acceptable to both patients and staff (referrers from within the service, clinicians trained to facilitate the intervention, administrative staff) (Woolf et al., under review), in accordance with implementation science methodology, a full-scale feasibility randomised controlled trial is now underway to produce additional feasibility outcomes and data regarding efficacy and health/cost analyses, with preliminary data indicating considerable cost savings for the 10-week neuropsychological intervention (estimated at AUD 600 per patient) compared to traditional CBT programs (costed at AUD 2000 per patient) for this cohort (Woolf et al., under review). Thus, even where clinical benefit, acceptability and potential economic savings are immediately apparent, the process of implementation requires extensive and often resource-intensive commitment to demonstrating sustainable embedment within the service.

In a different kind of implementation initiative, Kinsella and colleagues (2020) worked with a consumer-advocate organisation (Dementia Australia) to support their staff to deliver a memory group intervention to their client group. In that instance, neuropsychologists provided specialist leadership, training, and consultation to support the implementation. This allowed much wider access to the neuropsychological intervention than would have been possible if neuropsychologists were directly funded to conduct the intervention. This example highlights the general issue of limited availability of specialist neuropsychology services, which in Australia tend to be concentrated within metropolitan areas, thus critically reducing the ‘reach’ (see Peters et al., 2013a, b) of both diagnostic and intervention services especially for geographically, socially, or financially isolated individuals. While the COVID-19 pandemic and associated public health restrictions have undoubtedly broadened the use of telehealth for remote delivery of clinical services and this is likely to be helpful for expanding access, not all neuropsychological interventions are amenable to remote or digital delivery.

These examples demonstrate that it is possible to enhance clinical implementation of evidence-based neuropsychological interventions into health services if clinicians, researchers, health service managers, and consumers work together towards the common goal of improving access to these interventions. It is important to recognise, however, that few clinical neuropsychologists have training or experience in service implementation. To achieve progress in this field, neuropsychologists are likely to require training, mentoring, and collaboration with experts in service implementation, project management, and change management. This is greatly enhanced when formal organisations provide a cohesive and structured pathway for collaboration. For example, in the older adult cohort, the federally-funded Australian Dementia Network’s core ‘Memory Clinic Initiative’ has a key goal to enhance and harmonise post-diagnostic support for older people with cognitive impairment in memory clinic settings across Australia, with a dedicated sub-group currently working to provide targeted professional development and training, peer support, and representation of patient and health professional (including neuropsychologists’) needs at the policy level. For more information on access to resources and opportunities to become involved, see: https://www.australiandementianetwork.org.au/.

In addition to several common implementation framework examples referenced above and key strategies provided in Box 1, additional guides covering key principles and practical examples are also provided in the ‘Resources’ section.

## Conclusions and Future Directions

**Fig. 5:**
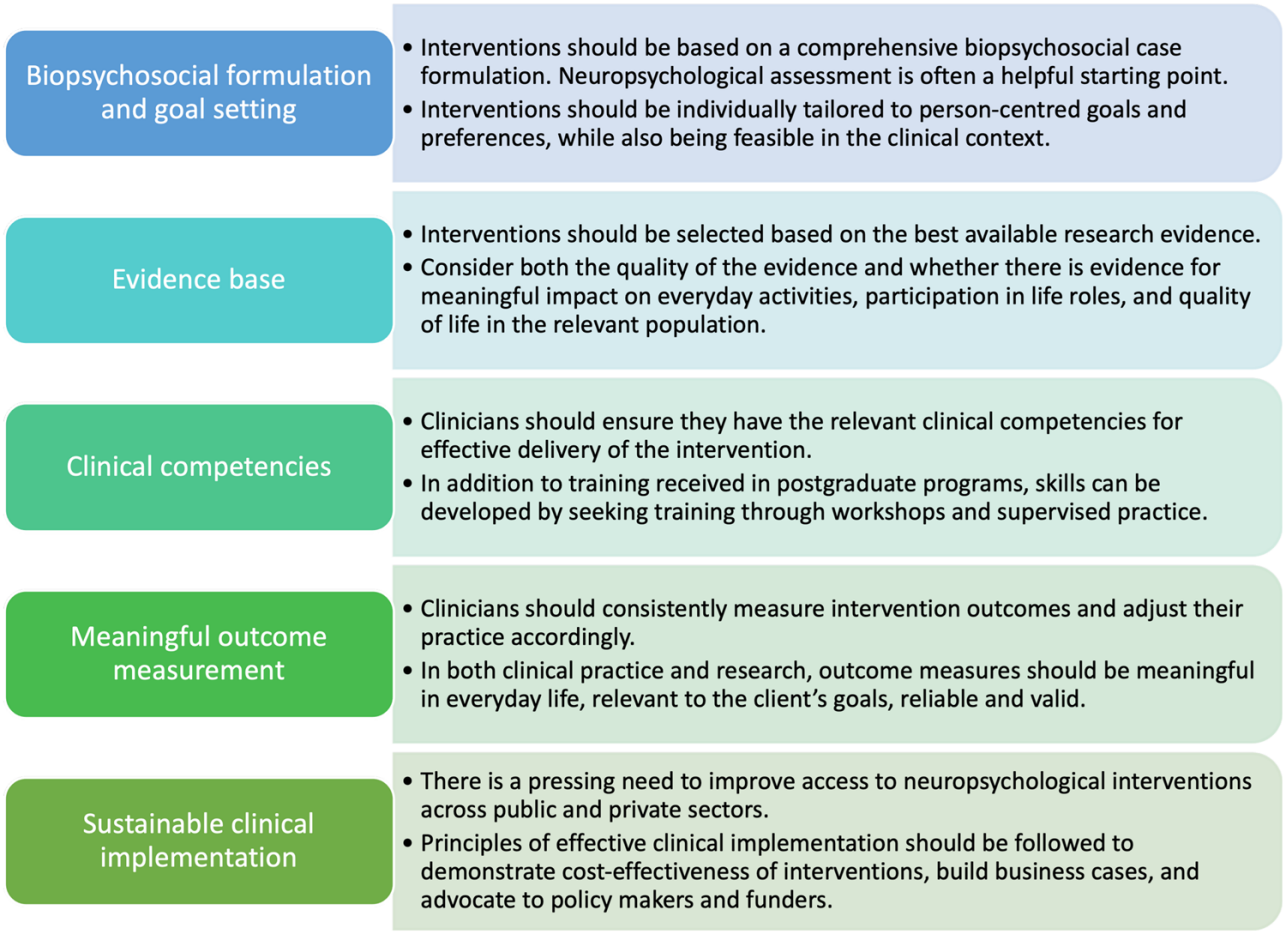
Summary of key considerations and recommendations from the working group

Figure 5 summarises the key considerations and recommendations put forward in this clinical guidance paper.

As a profession, we now need to progress in several important future directions.

### Building a Clinically Meaningful Evidence Base

There have been major and exciting developments in our understanding and application of neuropsychological interventions. However, there also remain key gaps in our knowledge and skill base, and there are several clear directions that would take us forward.

Firstly, more level 1 evidence is needed for many neuropsychological interventions. In particular, consistent inclusion of measures of activity and participation outcomes is crucial. The continued reliance on measures of objective cognitive impairment as primary outcomes has hampered ongoing development in the field and limited the impact of research on the everyday lives of people affected by brain conditions. The development of core outcome sets to allow better comparison of the everyday impact of various interventions would help progress towards this aim, such as those developed for TBI (Honan et al., 2017). Additionally, the development of rigorous trial designs that better reflect clinical practice (e.g. allocating participants to groups based on their biopsychosocial formulation and goals and randomising within those groups, rather than randomly allocating participants to treatments for which they are not suited) in combination with high-quality single case experimental designs will be important as we move towards an era of ‘precision rehabilitation’. Greater collaboration across the world will undoubtedly be necessary to achieve this, given the challenges with recruiting large samples for clinical trials with cohorts with neuropsychological conditions. Ultimately, a greater focus on designing research that is both methodologically sound *and* clinically relevant will facilitate smoother and more rapid translation to clinical practice.

Furthermore, evaluation of holistic interventions that combine cognitive, psychological, and behavioural elements (again, aligning more closely with clinical practice) is urgently needed. Related to this, the move towards evaluation of transdiagnostic, process-based psychological interventions would also help take the field beyond ‘siloed’ diagnosis-specific clinical trials. Integrated, multidisciplinary, transdiagnostic interventions enable a greater focus on clients’ goals, values, and preferences and provide opportunities for collaborative care and management of difficulties from multiple perspectives; however, rigorous research of such multifaceted interventions is often seen as costly (time, resources, training, collaboration) and may be vulnerable to criticism around an inability to define the ‘active ingredient’. Nonetheless, the benefits of such integrated care programs are currently being explored in both the ABI and dementia space. In ABI, holistic interventions that combine cognitive, emotional, and behavioural components are increasingly being evaluated, with promising results (e.g. Sathananthan et al., 2021). In dementia, a variety of neuropsychological interventions are being incorporated to add value to broader programs of person-centred, goal-oriented care, often sitting alongside complementary and well-established approaches including reablement, function-focused (a.k.a. restorative) care, and occupational therapist-led interventions (see Jeon et al., 2022). An example within the Australian context is the Interdisciplinary Home-based Reablement Program (I-HARP) currently under evaluation across five hospital and community-based sites within NSW (Jeon et al., 2019). This program uniquely recognises the key contribution of evidence-based neuropsychological techniques such as errorless learning and compensatory strategy training towards functional outcomes, within and alongside the more traditional home-based occupational therapy and nursing services which have previously been emphasised in other multidisciplinary programs, e.g. COPE (Clemson et al., 2018).

Finally, there is an unacceptable dearth of evidence for, and access to, culturally safe neuropsychological interventions for people from culturally and linguistically diverse backgrounds. This is particularly the case for indigenous Australians. Clinicians and researchers must act to address this gap as a matter of urgent priority.

### Embracing Telehealth and Other Technologies

In our current COVID-affected world and beyond, the use of telehealth and other technologies such as smartphones, virtual reality, and wearable devices for intervention delivery has become more important than ever. Therefore, another priority for future research and practice is to grow the evidence base and clinical translation of technology-enabled neuropsychological interventions. Neuropsychological interventions that have been trialled successfully so far using telehealth delivery (that is clinician-facilitated, rather than fully online or self-guided) include cognitive (usually memory) rehabilitation following stroke (Lawson et al., 2020), TBI (Bergquist et al., 2009; Bourgeois et al., 2007), and mild cognitive impairment and dementia (Cotelli et al., 2019); psychological therapies for depression, psychological distress, posttraumatic symptoms, sleep quality, and fatigue in TBI cohorts (Ownsworth et al., 2018); psychosocial interventions for depression post-stroke (Laver et al., 2020); interventions for challenging behaviours following TBI (McDonald et al., 2019); improving emotional wellbeing and cognitive function in brain tumour survivors (Ozier et al., 2019); support for caregivers of individuals with TBI (Powell et al., 2016) and dementia (Williams et al., 2018); and group treatment for emotional regulation post-TBI (Tsaousides et al., 2014). We are aware of numerous trials currently underway to expand this evidence base. Given these interventions often require the development of new clinical competencies, training clinicians in telehealth delivery will be essential for widespread access. Detailed teleneuropsychology guidelines are now available to guide telehealth delivery of neuropsychological interventions (Stolwyk et al., 2022).

### Weaving Interventions into Our Core Skill Base and Professional Identity

This clinical guidance paper arose from a need to clearly characterise a practical, evidence-based approach to the delivery of adult and older adult neuropsychological interventions in Australia, on the background of a professional history steeped in the tradition of assessment. To effectively transform our professional identity and clinical impact such that interventions are seen by both ourselves and others as central to our profession, it will be crucial to ensure that comprehensive, effective, skill-based training that incorporates opportunities for observational and experiential learning is widely available — and not only to students and registrars, but also to the senior clinicians who may not have received adequate (or any) training previously. We encourage all clinical neuropsychologists to consider engaging in opportunities to continually expand and/or update their skills in intervention delivery, which has the potential to improve their practice and outcomes across all types of clinical settings. Currently, there are limited such opportunities available (Wong et al., 2021a), and so, creating more is also a pressing need.

## Call to Action

We urge clinicians, researchers, and educators reading this clinical guidance paper to ask themselves what they can do in response to the issues and recommendations raised, to help move the field forward so that neuropsychological interventions can be considered ‘core business’ for clinical neuropsychologists.

We would encourage you to consider the following actions:

1. Understand and apply the key principles of neuropsychological intervention delivery to maximise your effectiveness.
2. Familiarise yourself with the evidence — and collect your own (on an individual client level and/or group level if possible).
3. Fill competency gaps with professional development including workshops, short courses, and supervision — and by implementing new skills in clinical practice.
4. Generate neuropsychological assessment recommendations that include feasible, evidence-based interventions and ensure these are implemented wherever possible.
5. Advocate up the line:
  - For resources and funding to support staff who can deliver interventions in your work setting — talk to managers, submit business cases, and ask people with lived experience to let health services know that they want to access neuropsychological intervention services and how they feel this would add value to the overall service they receive.
  - By reporting the outcomes and ‘telling the story’ of your interventions to team members, referrers, managers, and policy-makers.
  - By encouraging students to incorporate intervention delivery into their clinical practicum activities.

## Resources

### Existing Cochrane Reviews and Other High-Quality Meta-analyses on Neuropsychological Interventions

- Allida, S., Cox, K. L., Hsieh, C. F., Lang, H., House, A., & Hackett, M. L. (2020). Pharmacological, psychological, and non-invasive brain stimulation interventions for treating depression after stroke. *Cochrane Database of Systematic Reviews*, *1*(1), CD003437. 10.1002/14651858.CD003437.pub4
- Allott, K., van-der-EL, K., Bryce, S., Parrish, E. M., McGurk, S. R., Hetrick, S., . . . Velligan, D. (2020). Compensatory interventions for cognitive impairments in psychosis: A systematic review and meta-analysis. *Schizophrenia Bulletin, 46*, 869-883. 10.1093/schbul/sbz134
- Amatya, B., Khan, F., & Galea, M. (2019). Rehabilitation for people with multiple sclerosis: An overview of Cochrane reviews. *Cochrane Database of Systematic Reviews 1*(1), CD012732. 10.1002/14651858.CD012732.pub2
- Bahar-Fuchs, A., Martyr, A., Goh, A.M., Sabates, J. & Clare, L. (2019). Cognitive training for people with mild to moderate dementia. *Cochrane Database of Systematic Reviews*, (3):CD013069. 10.1002/14651858.CD013069.pub2
- Baker C., Worrall L, Rose M, Hudson K, Ryan B, O’Byrne L. A systematic review of rehabilitation interventions to prevent and treat depression in post-stroke aphasia. *Disability and Rehabilitation.* 2018;40(16):1870-1892.
- Beedham W, Belli A, Ingaralingam S, Haque S, Upthegrove R. The management of depression following traumatic brain injury: A systematic review with meta-analysis. *Brain injury.* 2020;34(10):1287-1304.
- Chandler, M.J., Parks, A.C., Marsiske, M., Rotblatt, L.J., & Smith, G.E. (2016). Everyday impact of cognitive interventions in mild cognitive impairment: A systematic review and meta-analysis. *Neuropsychology Review*, *26*(3), 225–251. 10.1007/s11065-016-9330-4
- Chun H-YY, Newman R, Whiteley WN, Dennis M, Mead GE, Carson AJ. A systematic review of anxiety interventions in stroke and acquired brain injury: Efficacy and trial design. *Journal of Psychosomatic Research.* 2018;104:65-75.
- das Nair, R., Cogger, H., Worthington, E., & Lincoln, N. B. (2016). Cognitive rehabilitation for memory deficits after stroke. *Cochrane Database of Systematic Reviews*, (9).
- Ford ME, Groet E, Daams JG, Geurtsen GJ, Van Bennekom CAM, Van Someren EJW. Non-pharmacological treatment for insomnia following acquired brain injury: A systematic review. *Sleep Medicine Reviews.* 2020;50:101255.
- Gavelin, H.M., Lampit, A., Hallock, H., Sabatés, J. & Bahar-Fuchs, A. (2020). Cognition-oriented treatments for older adults: A systematic overview of systematic reviews. *Neuropsychology Review, 30*(2), 167–193. 10.1007/s11065-020-09434-8
- Gertler, P., Tate, R. L., & Cameron, I. D. (2015). Non-pharmacological interventions for depression in adults and children with traumatic brain injury. *Cochrane Database Syst Rev*(12), Cd009871. 10.1002/14651858.CD009871.pub2
- Goverover, Y., Chiaravalloti, N. D., O’Brien, A., & DeLuca, J. (2018). Evidenced based cognitive rehabilitation for persons with multiple sclerosis: An updated review of the literature from 2007-2016. *Archives of physical medicine and rehabilitation, 99*, 390-407.
- Gromisch, E. S., Fiszdon, J. M., & Kurtz, M. M. (2018). The effects of cognitive-focused interventions on cognition and psychological well-being in persons with multiple sclerosis: A meta-analysis. *Neuropsychological rehabilitation*, 1-20.
- Hill, N.T., Mowszowski, L., Naismith, S.L., Chadwick, V.L., Valenzuela, M., & Lampit, A. (2017). Computerized cognitive training in older adults with mild cognitive impairment or dementia: A systematic review and meta-analysis. *The American Journal of Psychiatry*, *174*(4), 329–340.
- Kambeitz-Ilankovic, L., Betz, L. T., Dominke, C., Haas, S. S., Subramaniam, K., Fisher, M., . . . Kambeitz, J. (2019). Multi-outcome meta-analysis (MOMA) of cognitive remediation in schizophrenia: Revisiting the relevance of human coaching and elucidating interplay between multiple outcomes. *Neuroscience And Biobehavioral Reviews, 107*, 828-845. 10.1016/j.neubiorev.2019.09.031
- Khan, F., & Amatya, B. (2017). Rehabilitation in multiple sclerosis: A systematic review of systematic reviews. *Archives of physical medicine and rehabilitation, 98*(2), 353-367.
- Klein, O. A., Drummond, A., Mhizha-Murira, J. R., Mansford, L., & dasNair, R. (2019). Effectiveness of cognitive rehabilitation for people with multiple sclerosis: A meta-synthesis of patient perspectives. *Neuropsychological rehabilitation, 29*(4), 491-512.
- Lampit, A., Heine, J., Finke, C., Barnett, M. H., Valenzuela, M., Wolf, A., . . . Hill, N. T. M. (2019). Computerized cognitive training in multiple sclerosis: A systematic review and meta-analysis. *Neurorehabilitation and neural repair, 33*(9), 695-706. 10.1177/1545968319860490
- Loetscher, T., Potter, K. J., Wong, D., & das Nair, R. (2019). Cognitive rehabilitation for attention deficits following stroke. *Cochrane Database of Systematic Reviews*, Issue 11. Art. No.: CD002842. 10.1002/14651858.CD002842.pub3
- Liu, K. P., Hanly, J., Fahey, P., Fong, S. S., & Bye, R. (2019). A systematic review and meta-analysis of rehabilitative interventions for unilateral spatial neglect and hemianopia poststroke from 2006 through 2016. *Archives of physical medicine and rehabilitation*, 100(5), 956-979.
- Merriman, N. A., Sexton, E., McCabe, G., Walsh, M. E., Rohde, D., Gorman, A., ... & Hickey, A. (2019). Addressing cognitive impairment following stroke: Systematic review and meta-analysis of non-randomised controlled studies of psychological interventions. *BMJ open*, 9(2), e024429.
- Mewborn, C.M., Lindbergh, C.A., Stephen Miller, L. (2017). Cognitive interventions for cognitively healthy, mildly impaired, and mixed samples of older adults: A systematic review and meta-analysis of randomized-controlled trials. *Neuropsychology Review,* 27(4), 403-439. 10.1007/s11065-017-9350-8
- Mowszowski, L., Lampit, A., Walton, C.C. & Naismith, S.L. (2016). Strategy-based cognitive training for improving executive functions in older adults: A systematic review. *Neuropsychology Review,* 26 (3), 252-270.
- Nijman, S. A., Veling, W., van der Stouwe, E. C. D., & Pijnenborg, G. H. M. (2020). Social cognition training for people with a psychotic disorder: A network meta-analysis. *Schizophrenia Bulletin, 46*(5), 1086-1103. 10.1093/schbul/sbaa023
- O’Donoghue, M., Leahy, S., Boland, P., Galvin, R., McManus, J., & Hayes, S. (2022). Rehabilitation of cognitive deficits poststroke: Systematic review and meta-analysis of randomized controlled trials. *Stroke*, 53(5), 1700-1710. 10.1161/STROKEAHA.121.034218
- Peppel LD, Ribbers GM, Heijenbrok-Kal MH. (2020). Pharmacological and non-pharmacological interventions for depression after moderate-to-severe traumatic brain injury: A systematic review and meta-analysis. *Journal of Neurotrauma*, 37(14):1587-1596.
- Rogers, J. M., Foord, R., Stolwyk, R. J., Wong, D., & Wilson, P. H. (2018). General and domain-specific effectiveness of cognitive remediation after stroke: Systematic literature review and meta-analysis. *Neuropsychology Review,* 28(3), 285-309.
- Shah, T.M., Weinborn, M., Verdile, G., Sohrabi, H.R. & Martins, R.N. (2017). Enhancing cognitive functioning in healthly older adults: A systematic review of the clinical significance of commercially available computerized cognitive training in preventing cognitive decline. *Neuropsychology Review,* 27(1), 62-80. 10.1007/s11065-016-9338-9
- Sherman, D.S., Mauser, J., Nuno, M., & Sherzai, D. (2017). The efficacy of cognitive intervention in mild cognitive impairment (MCI): A meta-analysis of outcomes on neuropsychological measures. *Neuropsychology Review*, *27*(4), 440–484. 10.1007/s11065-017-9363-3
- Sigmundsdottir, L., Longley, W. A., & Tate, R. L. (2016). Computerised cognitive training in acquired brain injury: A systematic review of outcomes using the International Classification of Functioning (ICF). *Neuropsychological rehabilitation, 26*(5-6), 673-741.
- Su Y, Yuki M & Otsuki M. Non-pharmacological interventions for post-stroke fatigue: Systematic review and network meta-analysis. *Journal of Clinical Medicine.* 2020;9(3).
- Sullivan KA, Kaye S-A, Blaine H, et al. Psychological approaches for the management of persistent postconcussion symptoms after mild traumatic brain injury: A systematic review. *Disability and Rehabilitation.* 2020;42(16):2243-2251.
- Taylor, L.A., Mhizha-Murira, J.R., Smith, L., Potter, K-J., Wong, D., Evangelou, N., Lincoln, N.B., & das Nair, R. (2021). Memory rehabilitation for people with multiple sclerosis. *Cochrane Database of Systematic Reviews, Issue 10,* Art. No.: CD008754. 10.1002/14651858.CD008754.pub4
- ten Brinke, L.F., Davis, J.C., Barha, C.K., & Liu-Ambrose, T. (2017). Effects of computerized cognitive training on neuroimaging outcomes in older adults: A systematic review. *BMC Geriatrics*, 17(1), 139. 10.1186/s12877-017-0529-x
- Vita, A., Barlati, S., Ceraso, A., Nibbio, G., Ariu, C., Deste, G., & Wykes, T. (2021). Effectiveness, core elements, and moderators of response of cognitive remediation for schizophrenia: A systematic review and meta-analysis of randomized clinical trials. *JAMA Psychiatry*. 10.1001/jamapsychiatry.2021.0620

### Suggested Further Reading

- Clare, L. (2008). *Neuropsychological rehabilitation and people with dementia*. Psychology Press. ISBN13: 9781841696768
- Lincoln, N.B., Kneebone, I.I., Macniven, J.A., & Morris, R.C. (2011). *Psychological management of stroke.* Wiley-Blackwell. ISBN13: 9780470684269
- Low, L., & Laver, K. (2021). Dementia rehabilitation: Evidence-based interventions and clinical recommendations. Australia: Academic Press.
- Peters, D. H., Adam, T., Alonge, O., Agyepong, I. A., & Tran, N. (2013a). Implementation research: What it is and how to do it. *British Medical Journal, 347,* f6753. 10.1136/bmj.f6753
- Peters, D.H., Tran, N.T. & Adam, T. (2013b). *Implementation research in health: A practical guide.* Alliance for Health Policy and Systems Research, World Health Organization. ISBN: 9789241506212
- Ponsford, J., Sloan, S., & Snow, P. (2013). *Traumatic brain injury: Rehabilitation for everyday adaptive living* (2nd Ed.). Psychology Press. ISBN13: 9781848720275
- Sohlberg, M.M. & Turkstra, L.S. (2011). *Optimizing cognitive rehabilitation: Effective instructional methods.* Guilford Publications. ISBN: 9781609182007
- Wilson, B.A., Gracey, F., Evans, J.J., & Bateman, A. (2009). *Neuropsychological rehabilitation: Theory, models, therapy and outcome.* Cambridge University Press. ISBN: 9780521841498
- Wilson, B., Winegardner, J., Van Heugten, C.M., & Ownsworth, T. (Eds). (2017*). Neuropsychological rehabilitation: The international handbook.* Routledge. ISBN: 9781138643093

### Resources for Clinicians

#### Websites

- CogTALE: https://cogtale.org
- NeuroBITE: https://neurorehab-evidence.com/web/cms/content/home
- ASSBI Resources: https://assbi.com.au/OnlineStore
- Australian Dementia Network: https://www.australiandementianetwork.org.au/clinician/resources/
- SharpBrains Brain Training Evaluation Checklist: https://sharpbrains.com/resources/10-question-evaluation-checklist

#### Communities of Practice

- BRAINSPaN: https://assbi.com.au/BrainSPan (Australia, NZ, Asia–Pacific).
- International Neuropsychological Society – Neuropsychological Intervention Special Interest Group (INS NI-SIG) (Worldwide).
- Neuropsychologists in Rehabilitation Consultation Group (Victoria, Australia).
- Victorian Neuropsychologists in the Community (VicNIC).
- Neuropsychological Rehabilitation Interest Group (Western Australia).
- Special Interest Group for Neuropsychological Intervention (NSW, Australia).

#### Short Courses

- https://www.latrobe.edu.au/courses/short-courses/adapting-psychological-therapies-for-people-with-cognitive-impairment

- https://www.latrobe.edu.au/courses/short-courses/cognitive-rehabilitation

## Figures and Tables

**Table 1:**
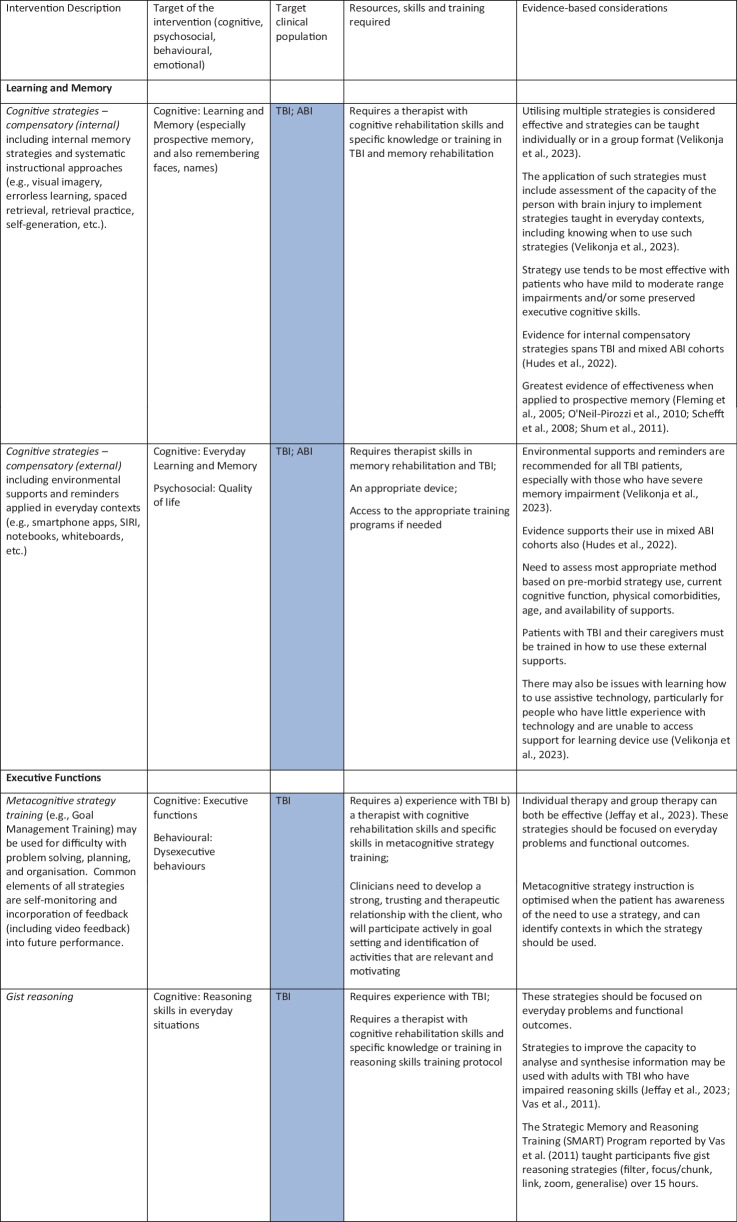
Interventions for which we have sound evidence of impact at the level of activity/participation — ABI

Blue shading indicates that the intervention and evidence applies to TBI only

**Table 2:**
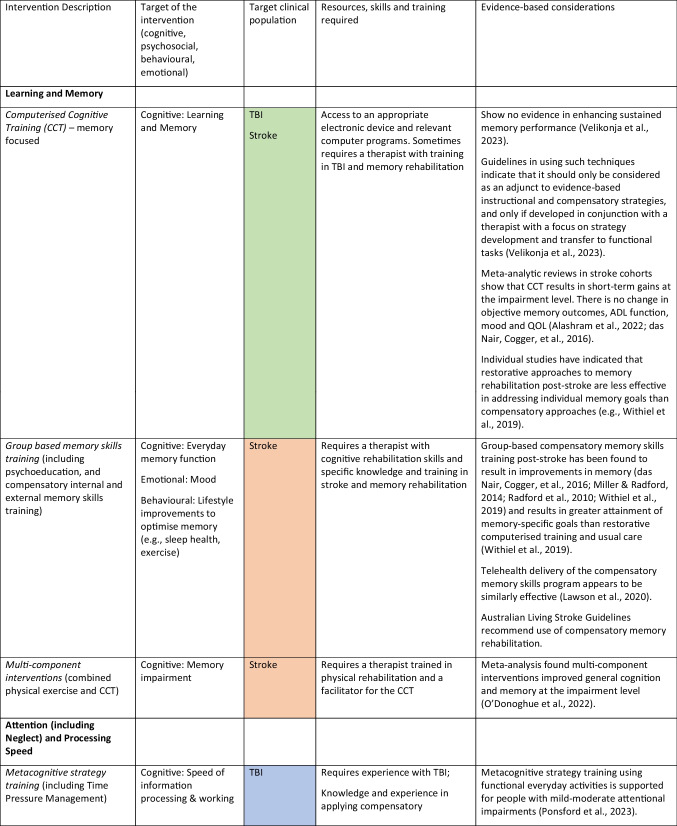

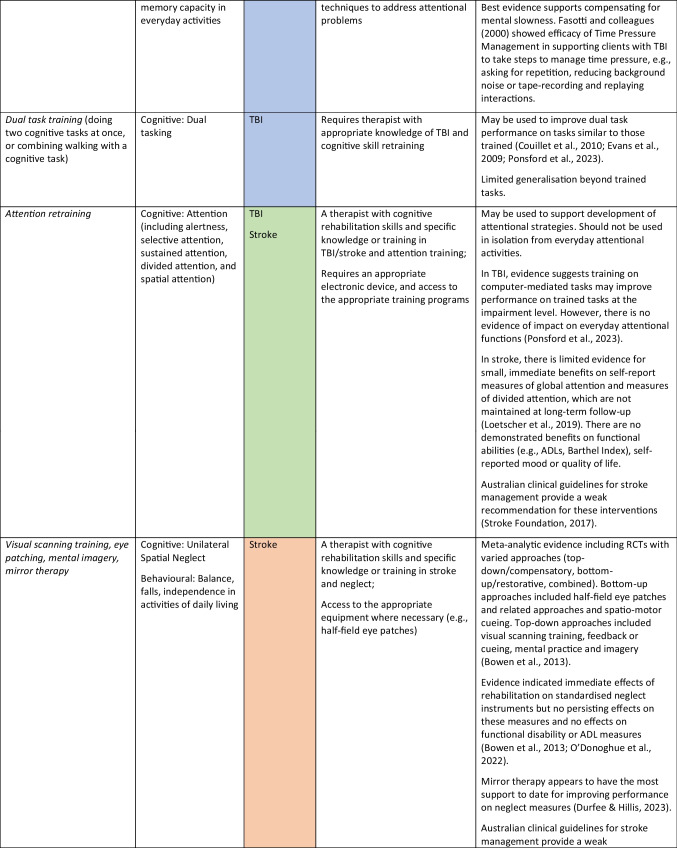

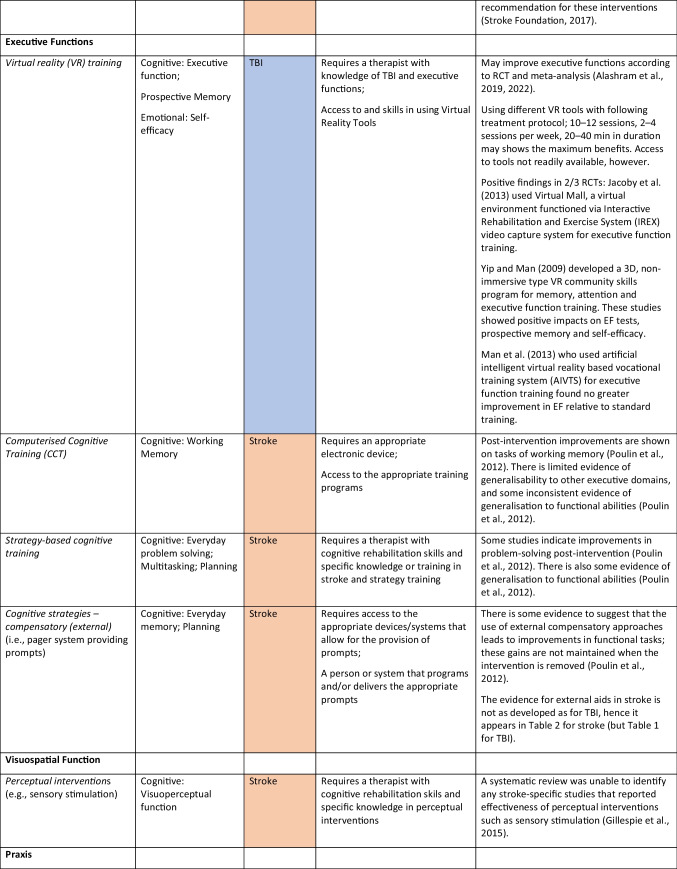

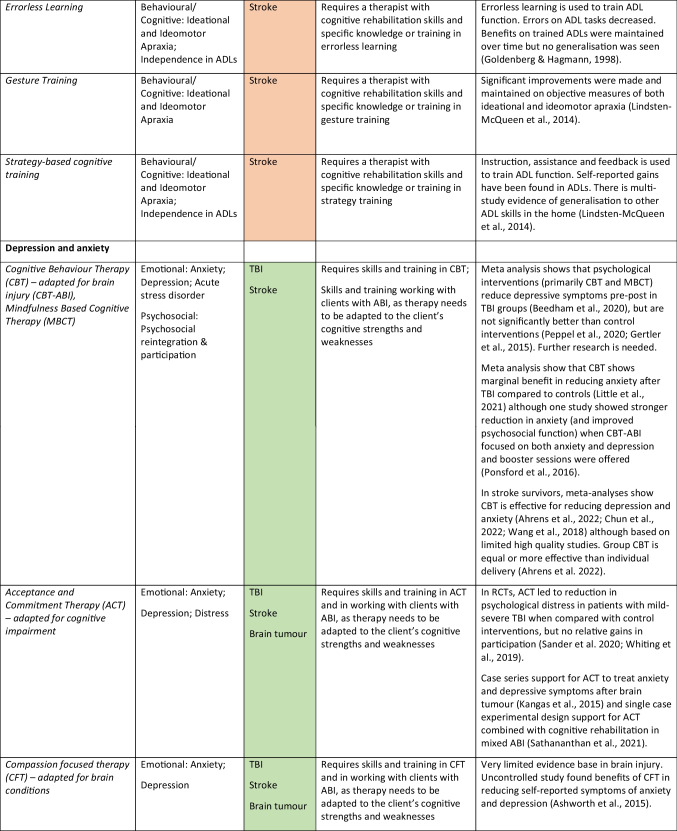

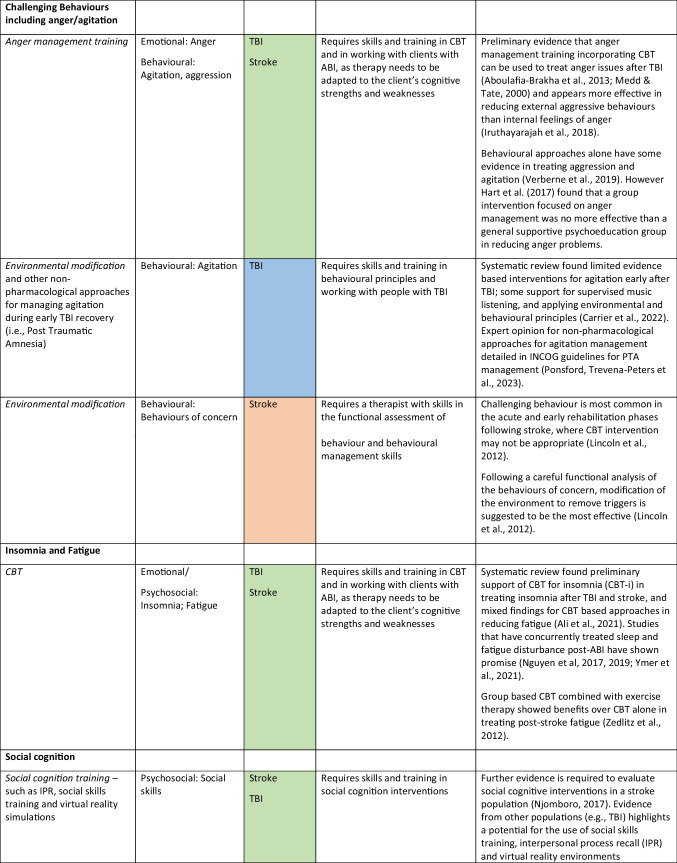

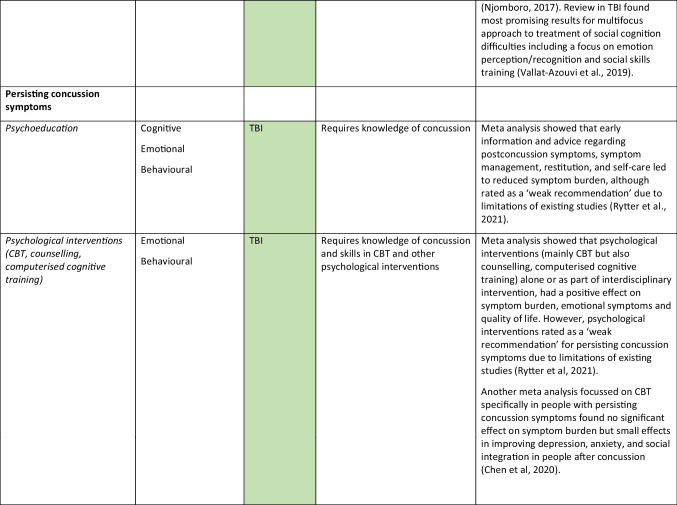
Interventions for which sound evidence of impact on activity/participation is still required (i.e. evidence is at level of impairment only OR only limited evidence of impact on activity/participation) — ABI

Blue shading indicates that the intervention and evidence applies to TBI only. Apricot shading indicates that the intervention and evidence applies to stroke only. Green shading indicates that the intervention and evidence applies to multiple forms of ABI

## Data Availability

No data was generated or analysed for this paper.

## Appendix 2. Tables 3 and 4 (summary of evidence in MS)

**Table 3:** Interventions for which we have sound evidence of impact at the level of activity/participation — multiple sclerosis (MS)

Intervention description	Target of the intervention (cognitive, psychosocial, behavioural, emotional)	Target clinical population	Resources, skills and training required	Evidence-based considerations
*Modified Story Memory Technique (mSMT)*, also called Kessler Foundation–modified Story Memory Technique	Cognitive: learning and memory Emotional: mood Behavioural: apathy	Multiple sclerosis (MS)	Therapist with general cognitive rehabilitation skills; Knowledge of MS is required	Has been found to benefit memory performance, self-reported everyday memory and general contentment, and family report of apathy and executive dysfunction (Chen et al., 2021; Chiaravalloti et al., 2013; Goverover et al., 2008, 2018b; Kalb et al., 2018; Taylor et al., 2021) This is recommended as a Practice Standard in MS (Goverover et al., 2018a, b)
*Self-generation*	Cognitive: learning and memory Behavioural: self-regulation Emotional: mood symptoms Psychosocial: quality of life	Multiple sclerosis (MS)	Therapist with general cognitive rehabilitation skills; Knowledge of MS is required	Benefits for memory performance, recall of activities of daily living, self-regulation, functional status, affective symptomatology, and quality of life (Basso et al., 2006; Chiaravalloti et al., 2005; Goverover et al., 2018a, b; Taylor et al., 2021) This is recommended as a practice option in MS (Goverover et al., 2018a, b)
*Visual imagery*	Cognitive: autobiographical memory	Multiple sclerosis (MS)	Requires a therapist with cognitive rehabilitation skills and specific knowledge or training in visual imagery	Has been found to improve autobiographical memory (Ernst et al., 2012, 2015; Goverover et al., 2018b; Impellizzeri et al., 2020; Taylor et al., 2021) This is recommended as a practice option in MS (Goverover et al., 2018a, b)

**Table 4:** Other interventions for which sound evidence of impact on activity/participation is still required (i.e. evidence is at level of impairment only OR only limited evidence of impact on activity/participation) — multiple sclerosis (MS)

Intervention description	Target of the intervention (cognitive, psychosocial, behavioural, emotional)	Target clinical population	Resources, skills, and training required	Evidence-based considerations
*Spaced retrieval*	Cognitive: new learning	Multiple sclerosis (MS)	Requires a therapist with cognitive rehabilitation skills and specific knowledge or training in spaced retrieval	This is recommended as a practice option in MS (Goverover et al., 2018b; Sumowski et al., 2010)
*Music mnemonics*	Cognitive: learning and memory	Multiple sclerosis (MS)	Requires a therapist with cognitive rehabilitation skills and specific knowledge or training in music mnemonics	This is recommended as a practice option in MS (Goverover et al., 2018b; Thaut et al., 2014)
*CCT* programs targeting attention (*attention process training* and RehaCom)	Cognitive: attention	Multiple sclerosis (MS)	Requires access to a computer (with a reliable internet connection for some online programs); A therapist with cognitive rehabilitation skills	Attention process training is recommended as a practice guideline, and RehaCom is recommended as a practice option (Goverover et al., 2018b)
*CCT* programs targeting multiple domains	Cognitive: Multiple cognitive domains	Multiple sclerosis (MS)	Requires access to a computer (with a reliable internet connection for some online programs); A therapist with cognitive rehabilitation skills	RehaCom (modules used were attention and concentration, plan a day, divided attention, reaction behaviour, and logical thinking) is recommended as a practice guideline (Goverover et al., 2018b)
*Cognitive strategies—restorative*	Cognitive: Memory span and ‘working memory’ (complex attention skills)	Multiple sclerosis (MS)	Some interventions require access to a computer (with a reliable internet connection for some online programs); A therapist with cognitive rehabilitation skills	These interventions include both paper-and-pencil type interventions and computerised interventions, usually lasting a few months, with 3–4 × 1 h intense training sessions per week (Dardiotis et al., 2018; das Nair et al., 2016b; Hamalainen & Rosti-Otajarvi, 2014; Lampit et al., 2019; Rosti-Otajarvi & Hamalainen, 2014; Sigmundsdottir et al., 2016)
*Multicomponent intervention* combining *computerised cognitive training* with holistic neuropsychological rehabilitation *(psychoeducation*, *compensatory cognitive strategies*)	Cognitive: verbal memory; processing speed; attention; executive function Emotional/psychosocial: depression; fatigue; quality of life	Multiple sclerosis (MS)	Requires access to a computer (with a reliable internet connection for some online programs); A therapist with general neuropsychological and/or broad cognitive rehabilitation skills; Knowledge of MS is required	Evidence for improvements in verbal memory, processing speed, attention and executive functions at the impairment level (das Nair et al., 2016b; Hamalainen & Rosti-Otajarvi, 2014; Sigmundsdottir et al., 2016) Improvements have been found in perceived cognitive functioning, use of compensatory strategies, and attainment of personal rehabilitation goals (Hamalainen & Rosti-Otajarvi, 2014)
*Multicomponent intervention* combining *metacognitive strategy training* with *mindfulness* and/or other cognitive rehabilitation	Cognitive: speed of information processing Emotional/psychosocial: cognitive concerns, achieving personalised goals, stress, fatigue	Multiple sclerosis (MS)	‘MaTiMs’, based on this research, is freely available to people with MS in the public health system in Germany	Some evidence for at least short-term improvements in cognitive concerns, in achieving personalised goals, in coping self-efficacy, stress, and fatigue (Nauta et al., 2023; Baetge et al., 2023; Pöttgen et al., 2015, 2022) Additional mindfulness training leads to longer-lasting improvements in speed of thinking. Effects tend to wane over time, so booster sessions are recommended

## Appendix 3. Table 5 (summary of evidence in psychiatric disorders)

**Table 5:** Interventions for which we have sound evidence of impact at the level of activity/participation — psychiatric disorders

Intervention description^a^	Target of the intervention (cognitive, psychosocial, behavioural, emotional)	Target clinical population	Resources, skills and training required	Evidence-based considerations
*Cognitive remediation therapy (CRT)* or *computerised cognitive training (CCT)*	Cognitive: CRT and CCT directly target cognition with either domain-specific or multidomain exercises, with goal of improving functioning (activity/participation)	Psychotic disorders, particularly schizophrenia Clinical high risk^b^ for psychosis Mood disorders^b^ Eating disorders, particularly anorexia nervosa^b,c^	Requires access to a computer and relevant cognitive training software, as well as basic computer proficiency (e.g. using a mouse and keyboard, running a program or opening a website); Some programs are web-based and can be completed individually at home with relatively remote therapeutic input; Requires task analysis of programs to determine cognitive skills required, pre-requisite skills, audio-visual experience, strengths and potential challenges	CRT is more effective when provided with strategy training/coaching from a trained therapist than pure drill-and-practice and self-administered approaches Effects appear similar for individual and group-based CRT and at varying training dosages. The high number of programs available can make it difficult for clinicians to navigate and choose which is most appropriate for their client base and practice More research is needed to understand the patient characteristics that predict response to CRT, such as age, premorbid or baseline ability, motivation, expectancy, maladaptive thinking styles, metacognition, and insight Systematic reviews and meta-analyses of CRT in schizophrenia show significant small-to-moderate improvements in cognition (see Bryce et al., 2016; Grynszpan et al., 2011; Kambeitz-Ilankovic et al., 2019; McGurk et al., 2007; Vita et al., 2021; Wykes et al., 2011). Effects on functioning are also significant and small-to-moderate. Much stronger effects on community functioning and participation are observed when CRT is provided in combination with psychosocial rehabilitation (e.g. supported employment) (Vita et al., 2021; Wykes et al., 2011)
*Compensatory cognitive strategies (*including *internal* and *external strategies, environmental supports,* and* errorless learning)*	Cognitive: Everyday cognitive function Psychosocial: Real world community functioning and functional capacity	Psychotic disorders, particularly schizophrenia	Intensive intervention but can be delivered flexibly; Many of the research-based compensatory interventions are manualised; Some compensatory interventions are delivered in the community and require environmental supplies to support functioning within the home and community (e.g. Cognitive Adaptation Training; Velligan et al., 2000)	Most compensatory interventions are delivered individually. One exception is Cognitive Compensatory Training which has been trialled in groups, but can also be delivered individually (Twamley et al., 2012) More research is needed to understand whether compensatory approaches are more effective when combined with other psychosocial interventions such as supported employment More needs to be understood regarding the characteristics of individuals most likely to benefit most from compensatory approaches Longer treatment length is associated with larger effect sizes for functioning outcomes (Allott et al., 2020) A systematic review and meta-analysis showed significant moderate effects on functioning (effect size = 0.46) post-intervention, with evidence of durability at follow-up (effect size = 0.36) (Allott et al., 2020). Effect sizes remained similar regardless of specific compensatory approach used. Improvements have also been observed in symptoms and quality of life
*Social cognitive training*	Cognitive/psychosocial: social cognitive training directly targets social cognition broadly or in specific targeted domains (emotion recognition, theory of mind, social perception, attributional style)	Psychotic disorders, particularly schizophrenia	Most interventions involve social cognitive stimuli such as videos, photographs, cartoons, or activity sheets; Audio-visual equipment and computers and software are often required to deliver social cognitive training; Social cognitive training is often delivered in groups, so a facility for running groups and multiple facilitators may be required	Duration, intensity, and breadth of the social cognitive training program does not appear to moderate the training effects Durability of effects remains unclear, as does the characteristics of individuals most likely to respond to social cognitive training While the evidence base for social cognitive training is less advanced than CRT, there is now robust evidence based on systematic reviews and meta-analyses significant moderate to large improvements in affect recognition, social perception, and theory of mind (Kurtz & Richardson, 2012; Kurtz et al., 2016; Nijman et al., 2020). There is less consistent evidence for the efficacy of social cognitive training in changing attributional style. Broad-based social cognitive training is associated with large improvements in functioning (effect size = 0.82), which is generally sustained at follow-up (effect size = 0.66) (Nijman et al., 2020)

^a^For simplicity types of intervention have been described separately, but many programs involve a combination of neuropsychological interventions

^b^Fewer studies have been conducted in these psychiatric conditions compared with schizophrenia; thus, the evidence is more limited to date (for reviews see Bowie et al., 2013; Danner et al., 2015; Glenthøj et al., 2017; Lindvall Dahlgren & Ro, 2014; Motter et al., 2016; Woolf et al., 2021)

^c^CRT has a specific focus on improving cognitive flexibility and global/contextual (‘bigger picture’) processing in eating disorders, due to the specific impairments observed in these domains

## Appendix 4. Tables 6 and 7 (summary of evidence in dementia)

**Table 6:** Interventions for which we have sound evidence of impact at the level of activity/participation — dementia

Intervention description	Target of the intervention (cognitive, psychosocial, behavioural, emotional)	Target clinical population	Resources, skills and training required	Evidence-based considerations
*Cognitive stimulation therapy —* techniques include reality orientation, reminiscence therapy, music and/or art therapy, validation therapy	Cognitive: global cognition Psychosocial:quality of life, wellbeing, communication, social interaction Emotional: mood	Mild-moderate dementia (mostly AD)	Resources include external orientation stimuli (e.g. orientation boards, calendars), instruments and/or audio equipment for music therapy, art/craft supplies for art therapy	Focuses on engagement in a range of activities and discussions, usually in a group format. Improvements have been shown in global cognition, using screening tools such as the MMSE, equating to an approximate 6-month delay in cognitive decline. Greater benefit if sessions occur > 1/week, and for people with mild dementia (Woods et al., 2023) Evidence for improvements in ADLs, quality of life, mood, communication, and social interaction, and decrease in challenging behaviours (Woods et al., 2023). Inferences in this body of work are limited by the lack of active control groups, and few follow-up studies No negative effects found (Woods et al., 2023)
*Cognitive rehabilitation* (also *cognitive strategies — compensatory*)	Behavioural: performance of functional tasks (i.e. ADLs, IADLs, social/community/study/vocational activities, etc.) Emotional: mood, satisfaction Psychosocial: quality of life, engagement, functional decline	Mild-moderate dementia (mostly AD)	Training and competency in collaborative goal-setting is essential for individualised, goal-oriented therapy; Requires competency in a variety of individual cognitive intervention techniques which can be utilised to address specific goals (e.g. cognitive strategies, environmental modification, psychoeducation)	Large, positive and sustainable effects have been shown across high quality studies for improvements in performance on goal-related activities, as rated by the person living with dementia as well as informants (Kudlicka et al., 2023) Reduced insight may be a barrier to recognising functional limitations, which in turn can impact goal-setting and engagement in the rehabilitation process Improvements have also been shown on trained, everyday tasks (e.g. recalling face-name associations, balancing checkbook — Tappen & Hain, 2014), cognitive performance (e.g. attention, memory; see Kudlicka et al., 2023) and in ratings of satisfaction, for both the person with dementia and their family carers ( Clare et al., 2019a, b, 2010), self-efficacy (Kudlicka et al., 2023), and quality of life (Kim, 2015) Some studies have shown sustained benefits, for example decreased dependence and caregiver burden during goal-related activities 1-year post-intervention (Germain et al., 2019), and reduced functional decline 2 years post-intervention (Amieva et al., 2016)

**Table 7:** Other interventions for which sound evidence of impact on activity/participation is still required (i.e. evidence is at level of impairment only OR only limited evidence of impact on activity/participation) — dementia

Intervention description	Target of the intervention (cognitive, psychosocial, behavioural, emotional)	Target clinical population	Resources, skills and training required	Evidence-based considerations
*Strategy-based cognitive training (SCT), computerised cognitive training (CCT)*	Cognition: global cognition as well as individual cognitive domains	Mild to moderate dementia (mostly AD, although language training is also used in primary progressive aphasia)	Repetitive, drill-and-practice training in specific cognitive domains (e.g. language or working memory) may require preparation of basic cues and stimuli such as word labels, pictures, and number series; SCT involves active teaching, modelling and guidance in adaptive techniques by a clinician facilitator. Resources may include external memory aids (e.g. notebooks, diary, post-it notes), stimuli (e.g. playing cards for working memory training), and preparation of templates (e.g. for problem-solving heuristics); CCT requires reliable access to a computer, cognitive training software, and/or the internet (for web-based programs) as well as basic computer proficiency (e.g. using a mouse and keyboard, running a program or opening a website)	The Cochrane systematic review and meta-analysis by Bahar-Fuchs et al. (2019) included 33 studies comprising > 2000 participants. Small-to-moderate improvements were seen in global cognition and semantic verbal fluency, compared to passive control conditions, both post-training and sustained over the medium term (i.e. 3–12 months). However, cognitive benefits did not extend to other domains and were not seen in comparison to active control conditions While there is insufficient evidence for benefits in non-cognitive outcomes (e.g. mood, daily functions), there was no evidence that cognitive training was associated with adverse outcomes for the person with dementia or caregivers Most participants across the 33 studies were community-based, indicating the feasibility of conducting cognitive training interventions in the community. The variety of studies demonstrated that training may be delivered in group or individual format, may target single or multiple domains, and may be augmented by other elements such as reality orientation Several systematic reviews and meta-analyses have explored the efficacy of either strategy-based or computer-based CT in dementia; however to our knowledge, none have completed a head-to-head comparison of the two. Moreover, even computer-based training may be clinician-mediated, making it even more difficult to tease apart these key components. Methodological heterogeneity is a key limitation across all reviews in this area; however, the most recent, high-quality Cochrane Review (Bahar-Fuchs et al., 2019) noted that exploratory subgroup analyses did not reveal strong differences across the various approaches
*Cognitive strategies — compensatory (internal)*	Cognitive: memory Behavioural: ‘challenging’ behaviours (e.g. verbal/physical aggression)	Mild-to-moderate dementia	Specific training is required for each technique; These strategies have few resource requirements as they primarily relate to the internal processes of learning or task implementation, however some aspects may require physical materials (e.g. signs or illustrations can be used as visual cues or prompts for procedural memory training)	Techniques include procedural memory training (Eslinger & Damasio, 1986; Sabe et al., 1995), dual cognitive support (i.e. support at both encoding and retrieval of information; Kinsella et al., 2007), and spaced retrieval (Camp, 2001; Clare et al., 2002), as reviewed by Creighton et al., 2013 Spaced retrieval has also been used to help manage challenging behaviours (e.g. verbal/physical aggression) which has clear relevance for participation/activity; however this application has not been explicitly measured Notably, utilising errorless learning to acquire these individual strategies appears to be key to their effectiveness
*Cognitive strategies — compensatory (external)* using technology	Cognitive: memory, executive functions (e.g. self-monitoring, initiation) Behavioural: wandering Emotional: loneliness, depression, anxiety Psychosocial: social interaction	All stages and subtypes of dementia	Requires acquisition and training of the person with dementia (and family/carers) in use and maintenance of the technology; Cost and availability of electronic devices may vary	External cognitive aids incorporing technology include electronic pill dispenser boxes, electronic diaries, wearable cameras (e.g. Silva et al., 2017), interactive ‘pet’ robots (e.g. Moyle et al., 2017; Yu et al., 2022), and tracking devices Technology provides novel methods for supporting people with dementia. Although these methods are promising, most existing studies are limited by small samples, unreliable technology, conduct outside the home environment, and outcomes unrelated to everyday function and quality of life (Fleming & Sum, 2014; King & Dwan, 2017; Van der Roest et al., 2017; Zamiri et al., 2021)
*Environmental modification* and design	Cognitive: orientation, memory, visuospatial functions, attention, executive functions Behavioural: wandering, navigating Psychosocial: social engagement, daily functioning	All stages and subtypes of dementia	Resources may range considerably depending on the scale of the modification, and typically involves deliberate selection of factors such as lighting, materials, paint (colour), texture, configuration, and temperature control. Consultation with other professionals such as occupational therapists and architects may be required	Modifications may include unobtrusive safety measures, varied ambience, single rooms, visual cues, and controlled stimulation levels (Fleming & Purandare, 2010) The ultimate environmental modification is the notion of ‘dementia villages’, with one implemented at Hogeweyk in the Netherlands, and others planned in Australia, the UK, and the USA. Rigorous trials on major environmental modifications are challenging to conduct, particularly due to issues with an appropriate control group, and a recent Cochrane review reported that there is currently insufficient evidence to draw conclusions about the impact of physical environment changes in residential care (Harrison et al., 2022)
*Psychoeducation*	Psychosocial: symptom management, patient safety, cost savings Emotional: adjustment to diagnosis; caregiver impact Behavioural: health and lifestyle activities	All stages and subtypes of dementia	Verbal discussion may be supplemented with written handouts, visual aids (e.g. diagrams or graphs); Delivery of the information may need to be modified for the audience (i.e. person with dementia vs. caregiver) in relation to format, length, language and complexity	Provision of psychoeducation should be based on up-to-date, evidence-based information relayed in an appropriate, accessible and understandable format Although direct evidence demonstrating benefits of neuropsychological feedback for people with dementia is still lacking, most people want to be told their diagnosis (Pinner & Bouman, 2003), although this should be established on an individual basis Benefits of diagnosing AD early include the opportunity for interventions, creating coordinated care plans, better symptom management, increased patient safety, cost savings, and delaying of institutionalisation (Dubois et al., 2016), while enabling the caregiver to adapt to the characteristic changes associated with dementia and their caregiver role, and feel more competent with fewer psychological issues (de Vugt & Verhey, 2013) In contrast, these benefits should be weighed for each individual against the issues of stigma, suicide risk, and the lack of disease-modifying therapies (Dubois et al., 2016) Clinically, many neuropsychologists provide psychoeducation regarding the strong evidence for health and lifestyle interventions, particularly exercise, in generally improving cognition in people with dementia (Livingston et al., 2020)
*Behavioural strategies* (e.g. caregiver educational and skills training; functional analysis of behaviour; activity planning; environmental modifications; caregiver support and self-care)	Behaviour: challenging behaviour, or behavioural and psychological symptoms such as agitation, aggression, disruption, shadowing, depression, repetitive behaviour, improve caregiver’s ability to manage Emotional: caregiver psychological health Psychosocial: caregiver quality of life, caregiver health,caregiver burden	Dementia	Requires skills and training in behaviour management and in working with clients with dementia, as tehcniques need to be adapted to the client’s cognitive strengths and weaknesses	Studies tend to use a wide variety of behaviour management techniques; often in combination, the specific effect of individual techniques is unclear (Brodaty & Arasaratnam, 2012; Moniz Cook et al., 2012; Tible et al., 2017)

## Appendix 5. Tables 8 and 9 (summary of evidence in MCI)

**Table 8:** Interventions for which we have sound evidence of impact at the level of activity/participation — MCI

Intervention description	Target of the intervention (cognitive, psychosocial, behavioural, emotional)	Target clinical population	Resources, skills and training required	Evidence-based considerations
*Strategy-based cognitive training* (SCT)	Cognitive: most studies in MCI have targeted learning and memory, while fewer have targeted attention, working memory and executive functions Emotional: self-efficacy, depression Behavioural: strategy use, daily functioning Psychosocial: quality of life, caregiver impact	MCI	SCT involves active teaching, modelling and guidance in adaptive techniques by a facilitator; Some teaching aids may be helpful, such as a whiteboard, worksheets, personalised templates or demonstration/practice with specific tools (e.g. a calendar, mobile phone, Dictaphone, Webster packs)	Alongside robust evidence for objective cognitive benefits (see Table 9), SCT results in increased strategy use and knowledge (Chandler et al., 2016; Kinsella et al., 2016; Reijnders et al., 2013) and is effective in improving memory self-efficacy and appraisal (Chandler et al., 2016; Li et al., 2011; Reijnders et al., 2013), self-reported depression, quality of life and day-to-day functioning (Li et al., 2011; WHO, 2019; Sanz Simon et al., 2012), and potentially also caregiver wellbeing (Chandler et al., 2016). Many studies have offered SCT within a multifaceted, group-based format where cognitive strategies are taught alongside health and lifestyle psychoeducation for dementia risk, and coping strategies (Anderson, 2019) Clinical judgement is required to determine appropriate strategies in the context of the client’s neuropsychological profile of strengths and weaknesses and their overall goals and motivations, (see (Giebel & Challis, 2015) Provision of health-related information alone is insufficient to induce behaviour change (Gardner et al., 2012). Alongside instruction and practice, SCT should ideally incorporate opportunities to demonstrate applicability to ‘real-world’ scenarios (generalisation), with clear opportunities for implementation (such as homework tasks to ‘trial’ various strategies and report back regarding success or challenges) (see Hampstead et al., 2014)
*Multimodal interventions*	Cognitive: global cognition and specific domains Behavioural: everyday functioning, engagement with lifestyle or health-related activities (e.g. physical exercise, dietary patterns, mindfulness) Emotional: depression, anxiety, stress Psychosocial: engagement in various activities, e.g. social, community, vocational, and study	MCI	Clinicians providing non-cognitive interventions should ensure that they are competent to deliver such interventions, or work with other allied health professionals (e.g. exercise physiologists) or medical specialists; Additional resources may be required for non-cognitive elements of the intervention, such as exercise equipment, relaxation aids (music, etc.), and refreshments for social groups	These interventions target a broad range of outcomes and may be synergistic (e.g. Huckans et al., 2013; Valenzuela & Sachdev, 2009) It is inherently difficult to isolate the ‘active ingredient’ in multimodal interventions and this has been a source of criticism. However, it has been suggested that multimodal interventions may be more effective because they simultaneously support both restoration of primary networks and compensation via recruited networks (see Sherman et al., 2017) Meta-analytical results indicate clear cognitive benefits following multimodal compared to single interventions (Salzman et al., 2022), but findings are mixed regarding improvements in ADLs, mood, quality of life, and memory self-efficacy, although combined cognitive and physical exercise training seems to be most promising (Chandler et al., 2016), with a recent meta-analysis suggesting that simultaneous or sequentially combined training has greater benefits than either type of training alone (Gavelin et al., 2020). Nonetheless, individual studies have shown improvements to daily functioning (Kurz et al., 2009; Londos et al., 2008), self-reported depression (Diamond et al., 2015; Kurz et al., 2009), quality of life (Londos et al., 2008), and sleep quality (Diamond et al., 2015) Client preferences, capacity, and motivations are important to consider here, as some clients may enjoy a holistic or varied approach, whereas others may appreciate a more delineated intervention type

**Table 9:** Other interventions for which sound evidence of impact on activity/participation is still required (i.e. evidence is at level of impairment only OR only limited evidence of impact on activity/participation) — MCI

Intervention description	Target of the intervention (cognitive, psychosocial, behavioural, emotional)	Target clinical population	Resources, skills, and training required	Evidence-based considerations
*Psychoeducation* and* feedback*	Psychosocial: quality of life, adjustment, guidance in health and lifestyle behaviours promoting brain health Emotional: anxiety, distress	MCI	Psychoeducation should be tailored to suit: (a) cognitive strengths and weaknesses (e.g. limiting the pace or volume of information for people with slowed processing speed or poor working memory); and (b) relevant, known risk or protective factors for dementia, particularly where these are modifiable (e.g. vascular risk factors, alcohol use, depression)	Feedback regarding MCI following neuropsychological assessment can be viewed as an opportunity for a single-session intervention, incorporating information regarding well-established health and lifestyle risk and protective factors for dementia (Grill et al., 2017; Livingston et al., 2020; Norton et al., 2014). Clinicians should be cautious, however, in deciding when the diagnosis of MCI is likely to be helpful versus when it may cause undue distress or may be unnecessary. A recent review highlights the importance of the clarity and quality of information regarding an MCI diagnosis in terms of the patients’ experience (Blatchford & Cook, 2020). Furthermore, even after positive impact of a diagnosis, access to follow-up support after an MCI diagnosis is important to minimise distress (Blatchford & Cook, 2020)
*Computerised cognitive training (CCT)*	Cognitive: specific domains, commonly memory but may also target attention, working memory, executive functions, visuospatial processing. CCT programs vary. For example, exercises used in training may be domain-specific or multidomain While the primary focus of CCT is on cognitive performance, several studies have measured non-cognitive outcomes including: Emotional: depression, anxiety, apathy Psychosocial: quality of life	MCI	Reliable access to a computer, cognitive training software, and the internet (for web-based programs); Basic computer proficiency (e.g. using a mouse and keyboard, running a program or opening a website); CCT also requires substantial participant investment in terms of time commitment for sustained practice, motivation (particularly when training is completed independently), and possibly also monetary costs for equipment, software or subscriptions	While a recent Cochrane review (Gates et al., 2019) was largely inconclusive due to heterogeneity in methodological rigour and design, other systematic reviews and meta-analyses have shown robust effects for improving cognitive performance in people with MCI. For example, small to moderate and statistically significant positive treatment effects have been shown in learning, memory and working memory in two recent meta-analyses (Hill et al., 2017; Zhang et al., 2019). However, CCT effects seem to be most consistent within cognitive domains that are directly trained, with limited research on the generalisability or sustainability of effects over time (Gates et al., 2019). Nonetheless, there is increasing evidence of neurobiological changes following cognitive training in people with MCI, including enhanced hippocampal neurochemistry, increased brain metabolism and task-related regional activation (see reviews by Belleville & Bherer, 2012; Suo & Valenzuela, 2012; ten Brinke et al., 2017) as well as increased functional connectivity between different brain regions (Hampstead et al., 2022) Importantly, several meta-analyses have also shown benefits for mood (depression, anxiety, possibly even apathy) and quality of life (Chandler et al., 2016; Hill et al., 2017), from studies of reasonable methodological quality. One meta-analysis (Chandler et al., 2016) reported no effects of CCT on daily functioning; however, this may reflect a ceiling effect, since people with MCI are known to perform well on most traditional (gross) tests of ADLs Further research is needed regarding the optimal ‘dose’ of CCT in MCI (i.e. frequency, intensity, duration of sessions) (Gates & Sachdev, 2014), and adherence to home-based CCT may vary widely over the long-term (Turunen et al., 2019). Based on research in healthy older adults (Lampit et al., 2014), it is possible that clinician-facilitated CCT may enhance motivation and compliance compared to independent CCT (e.g. web-based programs completed at home) While CCT is not associated with adverse events, the time and cost required may be viewed as burdensome (Gates et al., 2019) To promote impact at the level of participation/activity, CCT may be most effective as part of a broader intervention, incorporating goal-setting and other relevant approaches (e.g. psychoeducation, strategy training)
*Strategy-based cognitive training (SCT)*	Cognitive — memory, attention, processing speed, working memory	MCI	See Table 8	As with CCT, meta-analyses and systematic reviews have shown robust improvements in cognitive performance following SCT (e.g. Reijnders et al., 2013) as well as multimodal interventions (e.g. Salzman et al., 2022; Sherman et al., 2017). Benefits are most consistently shown in the domain of memory, although improvements in attention, processing speed and working memory have also been demonstrated CCT and SCT have also been consistently linked with increased brain activation, particularly in frontoparietal regions, and either increased or maintained connectivity — providing evidence for both compensatory and restorative neurobiological mechanisms of these interventions (see review by Miotto et al., 2018) It is widely noted that effect sizes and outcome measures vary widely and this may in part relate to methodological heterogeneity in terms of quality, intervention characteristics, participant characteristics, and control conditions. Despite these limitations, the WHO recognises at least small positive effects on cognition following cognitive training in older people with MCI (*Risk Reduction of Cognitive Decline and Dementia: WHO Guidelines*, 2019) and recommends this as a useful approach for reducing the risk of cognitive decline and/or dementia

## Appendix 6. Table 10 (summary of evidence in healthy older adults)

**Table 10:** Interventions for which sound evidence of impact on activity/participation is still required (i.e. evidence is at level of impairment only OR only limited evidence of impact on activity/participation) — healthy older adults

Intervention description	Target of the intervention (cognitive, psychosocial, behavioural, emotional)	Target clinical population	Resources, skills, and training required	Evidence-based considerations
*Psychoeducation* (group)	Emotional: psychological wellbeing	Subjective cognitive decline	Knowledge of models of cognition, age related changes to cognition, other impacts on cognition (especially mood); Group facilitation skills	Improvement in psychological wellbeing reported for people with SCD following group psychoeducation intervention (*g* = 0.40 (0.03 to 0.76); Bhome et al., 2018). Psychoeducation should focus on cognitive ageing, expectancy modification, and the link between cognitive difficulties and anxiety and stress (e.g. Bhome et al., 2018) Psychoeducation around models of cognition, normal changes with ageing, and modifiable risk factors for cognitive decline is often a key component of SCT as well
*Strategy-based cognitive training* (SCT)	Cognitive: learning and memory, executive functions most commonly targeted Emotional: self-efficacy, memory-related affect, wellbeing Behavioural: strategy use, daily functioning Psychosocial: quality of life	Healthy older adults or subjective cognitive decline	SCT involves active teaching, modelling and guidance in adaptive techniques (may be internal or external strategies) by a facilitator; Some teaching aids may be helpful, such as a whiteboard, worksheets, personalised templates or demonstration/practice with specific tools (e.g. a calendar, mobile phone, Dictaphone, Webster packs)	Overall objective and subjecive memory improves with SCT, sometimes in conjunction with psychoeducation, as well as a focus on meta-cognition and self-efficacy (e.g. Reijnders et al., 2013) Regarding memory strategy training, Hudes et al. (2019) performed a meta-analysis on self-reported measures, finding that interventions improve self reported memory ability, memory self-efficacy, strategy use, memory-related affect, psychological wellbeing, quality of life. They noted no effects on everyday function, but few studies had measured it Regarding strategy training for executive functioning, Mowszowski et al.’s (2016) systematic review demonstrated a moderate effeect size on objective measures following training, which was most commonly in inductive reasoning. Although few studies examined the impact on activities of daily living, some promising effects were reported. Similarly, there is promising evidence that effects may continue for months to years past training, but more research is needed Group-based SCT interventions may be more cost effective, less resource intensive, and provide an opportunity for social interaction, which may allow for peer-to-peer support (West et al., 2008), resulting in improved self-efficacy and normalisation of age-related cognitive changes (Kinsella et al., 2016, 2020; Matthews et al., 2020)
*Computerised cognitive training (CCT)*	Cognitive: specific domains, including memory, attention, working memory, executive functions, visuospatial processing. CCT programs vary. For example, exercises used in training may be domain-specific or multidomain Behavioural: Some studies have reported everyday functioning	Healthy older adults	Reliable access to a computer, cognitive training software, and the internet (for web-based programs); Basic computer proficiency (e.g. using a mouse and keyboard, running a program or opening a website); CCT also requires substantial participant investment in terms of time commitment for sustained practice, motivation (particularly when training is completed independently), and possibly also monetary costs for equipment, software or subscriptions	Overall, small, but significant, effect sizes are found for cognition in general (e.g. Lampit et al. (2014) reported an effect size of Hedges’ *g* = 0.22 (0.15 to 0.29)). Change in specific domains is variable, with speed and visual memory most commonly improved, and executive functions often reported not to improve (e.g. Lampit et al., 2014; Shao et al., 2015; Tetlow & Edwards, 2017). Nevertheless, a recent meta-analysis focused on CCT specifically targeting working memory, cognitive flexibility, or inhibition found large effect sizes on targeted tasks (Nguyen et al., 2019a, 2019b). Training that is adaptive is more successful (Lampit et al., 2014; Nguyen et al., 2019a, b), as is supervised, rather than at-home training (Lampit et al., 2014). Small effect sized near transfer to tasks within the same domain, as well as transfer to cognitive tasks from different domains, has been reported (Nguyen et al., 2019a, b). Tetlow and Edwards (2017) report a significant effect of far-transfer to self-reported everyday function, although not to quantitative measures of everyday function
